# HMGA1 regulates trabectedin sensitivity in advanced soft-tissue sarcoma (STS): A Spanish Group for Research on Sarcomas (GEIS) study

**DOI:** 10.1007/s00018-024-05250-y

**Published:** 2024-05-17

**Authors:** David S. Moura, Jose L. Mondaza-Hernandez, Paloma Sanchez-Bustos, Maria Peña-Chilet, Juan A. Cordero-Varela, Maria Lopez-Alvarez, Jaime Carrillo-Garcia, Marta Martin-Ruiz, Pablo Romero-Gonzalez, Marta Renshaw-Calderon, Rafael Ramos, David Marcilla, Ramiro Alvarez-Alegret, Carolina Agra-Pujol, Francisco Izquierdo, Luis Ortega-Medina, Francisco Martin-Davila, Carmen Nieves Hernandez-Leon, Cleofe Romagosa, Maria Angeles Vaz Salgado, Javier Lavernia, Silvia Bagué, Empar Mayodormo-Aranda, Rosa Alvarez, Claudia Valverde, Javier Martinez-Trufero, Carolina Castilla-Ramirez, Antonio Gutierrez, Joaquin Dopazo, Nadia Hindi, Jesus Garcia-Foncillas, Javier Martin-Broto

**Affiliations:** 1 Health Research Institute-Fundación Jiménez Díaz University Hospital, Universidad Autónoma de Madrid (IIS-FJD, UAM), 28015 Madrid, Spain; 2 Institute of Biomedicine of Seville (IBIS, HUVR, CSIC, Universidad de Sevilla), 41013 Seville, Spain; 3 Clinical Bioinformatics Area, Fundación Progreso y Salud (FPS), CDCA, Hospital Virgen del Rocio, 41013 Seville, Spain; 4 Bioinformatics in Rare Diseases (BiER), Centro de Investigación Biomédica en Red de Enfermedades Raras (CIBERER), FPS, Hospital Virgen del Rocio, 41013 Seville, Spain; 5 Pathology Department, Son Espases University Hospital, 07120 Mallorca, Spain; 6 Pathology Department, University Hospital Virgen del Rocio, 41013 Seville, Spain; 7 Pathology Department, Miguel Servet University Hospital, 50009 Saragossa, Spain; 8 Pathology Department, Gregorio Marañon Universitary Hospital, 28007 Madrid, Spain; 9 Pathological Anatomy Service, Complejo Asistencial Universitario de León, 24071 Leon, Spain; 10 Pathology Department, Hospital Clinico San Carlos, 28040 Madrid, Spain; 11 Pathology Department, Ciudad Real General Hospital, 13005 Ciudad Real, Spain; 12 Pathology department, Canarias University Hospital, 38320 Santa Cruz de Tenerife, Spain; 13 Pathology department, Vall d’Hebron University Hospital, 08035 Barcelona, Spain; 14 Medical Oncology Department, Ramon y Cajal University Hospital, 28034 Madrid, Spain; 15 Medical Oncology Department, Instituto Valenciano de Oncologia, 46009 Valencia, Spain; 16 Pathology Department, Hospital de la Santa Creu i Sant Pau, 08025 Barcelona, Spain; 17 Pathology Department, Hospital Universitari i Politècnic la Fe, 46026 Valencia, Spain; 18 Medical Oncology Department, Gregorio Marañon Universitary Hospital, 28007 Madrid, Spain; 19 Medical Oncology Department, Vall d’Hebron University Hospital, 08035 Barcelona, Spain; 20 Medical Oncology Department, Miguel Servet University Hospital, 50009 Saragossa, Spain; 21 Hematology Department, Son Espases University Hospital, 07120 Mallorca, Spain; 22 INB-ELIXIR-es, FPS, Hospital Virgen del Rocío, 41013 Seville, Spain; 23 Medical Oncology Department, Fundación Jimenez Diaz University Hospital, 28040 Madrid, Spain; 24 General de Villalba University Hospital, 28400 Madrid, Spain; 25 Department of Oncology in University Hospital Fundación Jiménez Díaz,, Av. de los Reyes Católicos, 2, 28040 Madrid, Spain

**Keywords:** HMGA1, Soft-tissue sarcoma, Trabectedin, Leiomyosarcoma, mTOR pathway

## Abstract

HMGA1 is a structural epigenetic chromatin factor that has been associated with tumor progression and drug resistance. Here, we reported the prognostic/predictive value of HMGA1 for trabectedin in advanced soft-tissue sarcoma (STS) and the effect of inhibiting HMGA1 or the mTOR downstream pathway in trabectedin activity. The prognostic/predictive value of HMGA1 expression was assessed in a cohort of 301 STS patients at mRNA (n = 133) and protein level (n = 272), by HTG EdgeSeq transcriptomics and immunohistochemistry, respectively. The effect of HMGA1 silencing on trabectedin activity and gene expression profiling was measured in leiomyosarcoma cells. The effect of combining mTOR inhibitors with trabectedin was assessed on cell viability in vitro studies, whereas in vivo studies tested the activity of this combination. *HMGA1* mRNA and protein expression were significantly associated with worse progression-free survival of trabectedin and worse overall survival in STS. HMGA1 silencing sensitized leiomyosarcoma cells for trabectedin treatment, reducing the spheroid area and increasing cell death. The downregulation of HGMA1 significantly decreased the enrichment of some specific gene sets, including the PI3K/AKT/mTOR pathway. The inhibition of mTOR, sensitized leiomyosarcoma cultures for trabectedin treatment, increasing cell death. In in vivo studies, the combination of rapamycin with trabectedin downregulated HMGA1 expression and stabilized tumor growth of 3-methylcholantrene-induced sarcoma-like models. HMGA1 is an adverse prognostic factor for trabectedin treatment in advanced STS. HMGA1 silencing increases trabectedin efficacy, in part by modulating the mTOR signaling pathway. Trabectedin plus mTOR inhibitors are active in preclinical models of sarcoma, downregulating HMGA1 expression levels and stabilizing tumor growth.

**Supplementary Information:**

The online version contains supplementary material available at 10.1007/s00018-024-05250-y.

## Introduction

Soft-tissue sarcoma (STS) is a group of usually aggressive mesenchymal neoplasms that can affect adults and pediatric patients with a high mortality rate. For the treatment of STS, surgery with or without perioperative radiotherapy or chemotherapy is the backbone for localized tumors, while single-agent doxorubicin or doxorubicin-based therapy is the upfront systemic line for STS when chemotherapy is required. On the other hand, only a few treatment options have emerged in the past 20 years as second-line therapies for advanced STS, including trabectedin, pazopanib, gemcitabine combinations, and eribulin [1–3]. This latter has only been approved for patients with advanced liposarcomas. However, the lack of new therapeutic options is only one of the many issues that STS patients have to face daily. In fact, the lack of predictive biomarkers of response for first- and second-line drugs in advanced STS, is also an unmet need. The search for predictive biomarkers, either at a simple level (with single genes/proteins) [4] or at a more complex level with molecular signatures [5, 6], may improve survival through a better selection of patients while avoiding potential toxicities from ineffective therapies [7–9].

Epigenetic reprogramming regulation, which impacts the transcriptional program of downstream genes leading to altered phenotypes, tumor progression, and drug resistance, is a topical issue in sarcoma research [10, 11]. Epigenetic regulation occurs at multiple levels and it may involve histone post-translational modification writers [12], DNA methylation [13], and chromatin remodeling complexes [14, 15], as well as structural epigenetic chromatin factors proteins, such as the high-mobility group (HMG) proteins (e.g. High-Mobility Group AT-Hook 1: HMGA1) [16, 17]. Proteins participating in these regulatory epigenetic processes are potential prognostic and/or predictive factors in STS.

HMGA1 is a chromatin remodeling protein that binds to chromatin-AT-rich regions, in the minor groove, through the AT-hook DNA-binding domain, allowing access of other transcription factors to DNA [18]. HMGA1 is abundantly found in the nucleus and it has been implicated in numerous malignant processes, such as cell proliferation [19], invasion, migration and metastasis [20], transcriptional dysregulation [21], unpaired DNA damage repair mechanisms [22] and cancer stem cell (CSC) maintenance [23, 24]. *HMGA1* is normally overexpressed in cancer and its high levels have been associated with tumorigenic processes in various tumors, including STS [25–27]. In liposarcoma, HMGA1 overexpression was associated with trabectedin resistance, through a mechanism dependent on the activation of NFkB activity [26]. Trabectedin is a chemotherapeutic agent that has been approved for the treatment of adult patients with advanced STS, after failure of anthracyclines and ifosfamide, or for those who are unsuited to receive these agents [1]. As the main mechanism of action, trabectedin has been described to bind to the minor groove of DNA, forming trabectedin-DNA adducts, which interferes with oncogenic gene transcription [28]. Noteworthy, HMGA1 seems to also bind to the minor groove of the DNA [29], promoting the assembly of enhanceosome and gene transcription. However, the overexpression of HMGA1 has been also correlated with increased activity of trabectedin on thyroid and colon carcinoma cells, suggesting that the prognostic of HMGA1 may depend on the type of malignancy [30].

In this study, we evaluated the value of HMGA1, as well as other HMG proteins, as prognostic and/or predictive factors for trabectedin activity in a retrospective series of 301 patients with advanced STS. Additionally, preclinical research aimed to describe the mechanisms governed by HMGA1 protein that could affect trabectedin sensitivity.

## Material and methods

### Patients

Retrospective and prospective series of advanced STS patients treated with trabectedin in 18 hospitals from GEIS (Spanish Group for Research on Sarcoma) were considered. Formalin-fixed paraffin-embedded (FFPE) tumor samples were used in this study. Patients’ clinical data was collected and was accurate in a query-based task. Informed consent was obtained from all prospective participants and from a retrospective cohort that was followed up. All the procedures were performed according to national regulations, and the study protocol was approved by the Ethics Committees and the institutional review board of each participant center.

### Gene expression

The mRNA expression of *HMGA1*, *HMGA2*, *HMGB1*, *HMGB2,* and *HMGB3* was determined in tumor blocks, using the Oncology Biomarker Panel of HTG Molecular (HTG Molecular Diagnostics, Tucson, AZ, USA). This panel measures the expression of 2549 human mRNAs (https://www.htgmolecular.com/assays/obp) related to tumor biology. For gene expression assessment, the tumor blocks had at least 70% of viable tumor area. The libraries for RNA‐Seq were synthesized through HTG EdgeSeq chemistry, following manufacturers’ instructions [6]. Libraries were sequenced in a NextSeq 550 system (Illumina, San Diego, CA, USA). A FASTQ file was retrieved per sample from the sequencer and they were then aligned and parsed in the HTG EdgeSeq host software, obtaining a read counts matrix. The HTG EdgeSeq was run in the VERIP service laboratory of HTG. The data normalization of the complete set of genes was performed with trimmed mean of M‐values method (TMM), using the edgeR package from R/Bioconductor and adjusting it for the total reads within a sample [31].

### Immunohistochemistry

HMGA1 and HMGB1 protein expression assessment was carried out in FFPE samples. For tissue microarrays (TMAs) the tumor areas were selected by pathologists, who were blinded to clinical data. The TMA was built using a manual tissue microarrayer (Model MTA-1, Beecher; Sun Prairie, WI, USA), sampling 1.0 mm diameter cores from tumor blocks, corresponding to each included case. Four-μm sections were cut from the TMAs and stained with hematoxylin and eosin and with immunohistochemistry staining. Immunohistochemistry used an anti-HMGA1 rabbit monoclonal antibody (ab129153; Abcam, Cambridge, UK) or an anti-HMGB1 rabbit polyclonal antibody (ab18256; Abcam). Protein expression was evaluated as negative (0–4% of cells stained), + (5–24% of cells stained), +  + (26–49% of cells stained) and +  +  + (more than 50% of cells stained). The strength of the immunohistochemistry was evaluated in 3 levels: negative, weak, and strong. Protein expression was further grouped as low (< 50% positive cells) vs. high (≥ 50% positive cells) extension, whereas the strength of immunohistochemistry was grouped as having negative/weak intensity or strong intensity. A histoscore was calculated by multiplying protein expression and the strength of the immunohistochemistry levels. Two pathologists (RR and DM), with great expertise in sarcomas, were responsible for the protein expression review, blinded to clinical data.

### In vitro studies

#### Cell culture

For preclinical research the following STS cell lines were used: Angiosarcoma primary cell line ICP059 (established in-house from a female patient diagnosed with a grade 3 angiosarcoma of extremety), fibrosarcoma cell line HT-1080 (ATCC^®^ CCL-121^™^; ATCC, Old Town Manassas, VA, USA), leiomyosarcoma primary cell lines AA (kindly provided by Dr. Amancio Carnero of the Institute of Biomedicine of Seville, Seville, Spain) and CP0024 (established in-house from a female patients diagnosed with a grade 2 leiomyosarcoma of extremety), well-differentiated liposarcoma cell lines 93T449 (ATCC^®^ CRL-3043^™^; ATCC), malignant peripheral nerve sheath tumor (MPNST) primary cell line ICP060 (established in–house from a male patients diagnosed with a grade 3 MPNST of trunk wall) and sarcoma cell line SW982 (ATCC^®^ HTB-92^™^; ATCC). HT-1080 and AA cell line were maintained in F-10 medium (GibcoTM, Thermo Fischer Scientific, Waltham, MA, USA), ICP059, ICP060, 93T449 and CP0024 were cultured in RPMI cell medium (GibcoTM), and SW982 was cultured in Leibovitz’s L-15 Medium (GibcoTM) and all the cell culture mediums were supplemented with 10% FBS, 100 units/mL penicillin (PAA) and 100 μg/mL streptomycin. All these cell lines were maintained at 37 °C with 5% CO_2_. All the cell lines were routinely checked and tested for contamination by mycoplasma or fungi. All cell lines were discarded after 2 months of culture and new cells were obtained from frozen stocks.

#### Gene expression analysis

For determining *HMGA1* gene expression levels, by qRT-PCR, cells were cultured in 10 cm dishes for 48 h. Then, the cells were harvested and total RNA was isolated by the TRIzol^®^ (Invitrogen Corp., Carlsbad, CA, USA) -chloroform method, according to the manufacturer’s instructions. Reverse transcription was performed with 240 ng of RNA, using the High-Capacity cDNA Reverse Transcription Kit (Applied Biosystems^™^; Thermo Fischer Scientific), in the presence of MultiScribe^™^ Reverse Transcriptase and a random primer scheme for cDNA synthesis. Then, cDNA was amplified and quantified by qRT-PCR, using the GoTaq^®^ qPCR Master Mix Kit (Promega; Madison, WI, USA). Individual quantification of gene expression was performed using the comparative CT method (CT) and the expression was calculated as 2^−ΔCT^. The QPCR Human Reference was also included for relative expression against a universal RNA pool (Agilent, Santa Clara, CA, USA). The following assays were used for gene expression: *HMGA1* (Hs00852949_g1) and *GAPDH* (Hs03929097_g1). A total of three biological replicates, with three technical replicas each, were performed.

#### Western blot

For lysing cells and to extract protein RIPA buffer was used, adding a cocktail of protease and phosphatase inhibitors (Sigma-Aldrich). For the SDS-PAGE assay, a total of 20 µg/sample was loaded in the acrylamide gel. Proteins were transferred from the gel to 0.2 µm pore-size nitrocellulose membranes (Bio-Rad, Hercules, CA, USA). The membranes were then blocked for 1 h using 5% BSA (PanReac AppliChem, ITW Reagents) in 1X TBS-T (0.1% Tween20, Bio-Rad). After membrane blocking, the following antibodies were used for blotting proteins: anti-HMGA1 (ab129153; Abcam), anti-PARP1 (51-66396R; BD Bioscience, Franklin Lakes, NJ, USA), anti-cleaved Caspase 3 (#9661; Cell signaling, Danvers, MA, USA), anti-phospho-S6 (#4858; Cell signaling), anti-S6 (#2217; Cell signaling) and anti-α-tubulin (Sigma-Aldrich, T9026). Membranes were consequently incubated with Rabbit Anti-Mouse IgG–Peroxidase antibody (Sigma-Aldrich) or Goat Anti-Rabbit IgG H&L peroxidase-conjugated antibody (Abcam) secondary antibodies. For chemiluminescent detection, the HRP substrate was used. Image acquiring was performed using the ChemiDoc Imaging System (Bio-Rad). ImageJ was used to quantify protein expression levels.

#### HMGA1 gene expression silencing

To produce viral particles, the HEK293T cells were transfected with lentivirus-producing plasmids PMD2.G-VSV-G and pCMV-dR8.91 (Addgene, Watertown, MA, USA) and plkO.1-puro plasmid containing the sequence for either a non-targeting [SHC016-1EA (shControl), Sigma-Aldrich] RNA or containing HMGA1 shRNAs. Two shRNAs against HMGA1 were used [TRCN0000018949 (shHMGA1-446) and TRCN0000018951 (shHMGA1-281, Sigma-Aldrich], using 0.25 M CaCl_2_. To eliminate CaCl_2_ from the culture, a fresh DMEM medium was added to the HEK293T cells, 24 h after transfection. The lentivirus-containing medium was filtered through a 0.45 µm polysulfonate pore, complemented with 4 mg/ml polybrene (Sigma-Aldrich), 48 h after transfection, and the filtered medium was used to transduce CP0024 cells. Transductions were carried out every 12 h for a 36 h period. Puromycin (0.5 µg/ml) was used to select transduced cells for 10 days. HMGA1 silencing was verified by Western blot.

#### Affymetrix array and data analysis

Gene expression profiling was executed with Clariom^™^ S human assay (Applied Biosystems^™^; ThermoFisher Scientific, Inc.; Foster City, CA, USA) with RNA from 2D CP0024 cells. The following samples were used for the array: shHMGA1-281 and shHMGA1-446, as well as shControl (three biological replicas, pooling 3 experimental replicas each). Briefly, RNA was amplified and labeled using the GeneChip^®^ WT PLUS Reagent Kit (Thermo Fisher Scientific, Inc.; Waltham, MA, USA). The amplification used 100 ng of total RNA input, and it was performed following the procedures described in the WT PLUS Reagent Kit user manual. Then, cDNA was quantified, fragmented, and labeled to hybridize to GeneChip^®^ Clariom S Human Array (Thermo Fisher Scientific, Inc.), using 5.5 μg of single-stranded cDNA product and following the manufacturer protocols. Washing, staining (GeneChip^®^ Fluidics Station 450, Thermo Fisher Scientific, Inc.), and scanning (GeneChip^®^ Scanner 3000, Thermo Fisher Scientific, Inc.) were completed according to the protocols outlined in the user manual for cartridge arrays.

Arrays were background corrected and normalized using the RMA (Robust Multichip Average) method from the oligo (v1.54.1) package in R (v4.0.4). Probes were summarized and mapped to genes with the BrainArray annotation library (v25.0.0) for Entrez Gene IDs. Differential expression analyses were performed using linear models from the LIMMA (v3.46.0) package in R. Differences in gene expression were considered significant when FDR < 0.05 and log2 fold-change > 1 or < − 1. Gene Set Enrichment analyses were carried out using the clusterProfiler (v3.18.0) package in R with the gene sets provided by the Molecular Signature Database (v7.2). Only gene sets with a size between 5 and 500 were considered.

#### Spheroid formation

For spheroid formation, CP0024 (1.5 × 10^3^ cells/well) cells (transduced with HMGA1 shRNA or non-targeting shRNA) were seeded in Ultra-low-attachment 96-well plates (Corning; Corning, NY, USA). Images were captured after 8 days of 3D spheroid culture using an inverted microscope Olympus IX-71 (Olympus Corporation; Shinjuku City, Tokyo, Japan). At that moment (day 8), 3D cultures were treated with 10 nM trabectedin (PharmaMar, Madrid, Spain) for 72 h. Trabectedin was dissolved in DMSO. After removing trabectedin from the cell medium, images were acquired using an inverted microscope Olympus IX-71. The area of the spheroid was determined using the ImageJ tool Analyze Spheroid Cell Invasion In 3D Matrix (RRID: SCR_021204).

#### Flow cytometry

The levels of viable cells, apoptotic, early apoptotic, and necrotic cells were evaluated in the CP0024 cell line, transduced with shHMGA1 or shControl, and treated with 10 nM trabectedin for 24 h. FITC Annexin V Apoptosis Detection Kit with PI was used to determine cell death (Immunostep; Salamanca, Spain), following the manufacturer’s instructions. Three biological replicas were performed. Apoptosis levels were determined by flow cytometry (BD Accuri C6 Plus) and data was analyzed with BD Accuri C6 Plus software.

#### Cell viability assay

CP0024 and AA primary cell lines were seeded in 96-well plates and treated separately with increasing concentrations (1 × 10^−11^ M to 1 × 10^−8^ M) of trabectedin for 72 h. MTS assay (Promega) was used to evaluate cell viability. The concentrations inhibiting 50% of cell growth (IC_50_) were determined using nonlinear regression in Prism 5.0 (GraphPad Software; San Diego, CA, USA). To determine the effect of rapamycin treatment on the IC_50_ of trabectedin and in cell viability (MTS assay), cells (CP0024 and AA) were seeded in 96-well plates, pre-treated with 20 nM rapamycin for 2 h before adding trabectedin (1 × 10^−11^ M to 1 × 10^−7^ M) for 72 h. Combination index values were calculated using the median effect methods described by T-C Chou and P. Talalay [32] and the CalcuSyn software. For this determination, it was used MTS cell viability data of trabectedin (concentrations ranging from 1 × 10^−11^ M to 1 × 10^−8^ M) and rapamycin (concentrations ranging from 1 × 10^−10^ M to 1 × 10^−7^ M). Combination schemes were performed with a concentration ratio of trabectedin: rapamycin of 1:10.

Additionally, the protein expression of apoptotic markers (i.e. cleaved caspase 3 and PARP1) was determined by SDS-PAGE/western blot, after exposing 2D CP0024 cell cultures to 20 nM rapamycin and 2 nM trabectedin as single agents or in combination.

### In vivo studies

Five-week-old C57BL/6 J female mice were obtained from Envigo (Indianapolis, IN, USA) and an immunocompetent and highly aggressive fibrosarcoma-like model was induced by intramuscular injection of 3-methylcholanthrene [3-MC; 350 μg of 3-MC in 0.2 ml peanut oil (Sigma-Aldrich)] [33]. Tumors developed in 60 to 90 days and when they reached 150mm^3^ they were randomly assigned into four treatment groups: control (vehicle; n = 6), rapamycin (0.5 mg/kg; n = 7), trabectedin (0.15 mg/kg; n = 7), and rapamycin plus trabectedin (n = 7). Randomization was performed according to tumor volume size. Rapamycin was administered by intraperitoneal injection twice a week (days 0, 3, 7, and 10), whereas trabectedin was intravenous inoculated once a week on days 0 and 7. In combination treatments, rapamycin was given on the same days as described before, but trabectedin was administered once a week on days 1 and 8. The experiment lasted for 15 days. Tumor volume was measured with a caliper, and body weight was measured 3 times a week. The following equation was used to determine tumor volume: tumor volume = (length × width × depth)/2. The animals were sacrificed with CO_2_ at the end of treatment, and tumors were fixed with formalin and embedded in paraffin. For IHC studies, tumor samples were used to stain HMGA1 rabbit (1:10,000: ab129153; Abcam) and phospho-S6 rabbit (1:100; #4858; Cell signaling). Antigen retrieval was performed with a PT Link instrument (Agilent), using citrate buffer (pH 6.0) 97 °C, 20 min. 3,3-diaminobenzidine was applied to develop immunoreactivity for 5 min. The in vivo experiments were carried out following the ethical guidelines set by the Institutional Committee of Animal Research (Ethics committee of the university hospitals Virgen del Rocio and Virgen de la Macarena, Seville, Spain).

### Statistical analysis

Variables following binomial distributions, such as patient demographics were expressed as frequencies and percentages. Categorical variables were expressed as absolute and relative frequencies or as continuous variables as median and range (minimum–maximum). The log2 of gene expression levels were indicated as median and range. The U of Mann–Whitney nonparametric test was used to compare quantitative and qualitative variables. False discovery rate (FDR) was used to adjust for multiple comparisons. Both progression-free survival (PFS) and overall survival (OS), measured from trabectedin initiation, were estimated according to the Kaplan–Meier method, while the log‐rank test was used to access the prognostic value of the variables of interest (gene expression or protein expression and clinical outcomes). Patient radiological evaluation was performed every 8 weeks and the best response was considered at any time during patient treatment. Multivariate analysis was carried out according to the Cox proportional hazard regression model and it included the following variables: *HMGA1*, *HMGB1*, *HMGB2*, *HMGB3*, age, grade, and histology L-sarcoma (i.e. liposarcoma and leiomyosarcoma). Pearson’s r test was used to correlate HMG gene expression levels and HMGA1 gene and protein expression levels in cell lines. Student t-test was used to compare the effect of HMGA1 knockdown and/or trabectedin treatment in 3D spheroid areas and to compare mice tumor volumes across the different treatments in the in vivo study. The p‐values reported were 2‐sided, and the statistical significance was defined at p ≤ 0.05, except when mentioned differently. Statistical analyses were performed with SPSS 25.0 software (IBM, Armonk, NY, USA).

## Results

### Translational research

#### Patients’ demographics and treatment outcome

A total of 301 STS patients treated at any line of advanced disease with trabectedin were considered for this study. The median age of the study population was 51 years (range 14–79) with the female/male ratio being 53% (n = 159)/47% (n = 142). The most common subtypes were L-sarcomas with 167 cases (55%), whereas 134 (45%) patients were diagnosed with other STS histologies [e.g. undifferentiated pleomorphic sarcoma (UPS), synovial sarcoma and malignant peripheral nerve sheath tumor (MPNST), among others]. Sixty-four (21%) patients were metastatic at diagnosis. The histologic grade at the diagnostic time was as follows: 56% (n = 167) were grade 3, 34% (n = 70) were grade 2 and 13% (n = 39) were grade 1; this information was not available in 8% (n = 25) of the cases (Table 1).

**Table 1:** Patient demographics (N = 301)

	N
Gender	
Male	142 (47%)
Female	159 (53%)
Stage at diagnosis	
Localized	236 (78%)
Metastatic	64 (21%)
Not available	1 (1%)
Sarcoma subtype	
L-sarcomas	167 (55%)
Leiomyosarcoma	92 (31%)
Myxoid liposarcoma	37 (12%)
Dedifferentiated liposarcoma	22 (7%)
Well-differentiated liposarcoma	13 (4%)
Pleomorphic liposarcoma	3 (1%)
Non-L-sarcomas	134 (45%)
Synovial sarcoma	28 (9%)
Undifferentiated pleomorphic sarcoma	28 (9%)
Sarcoma NOS (not otherwise specified)	16 (6%)
Malignant peripheral nerve sheath tumor	15 (5%)
Other subtypes	47 (16%)
Grade	
1	39 (13%)
2	70 (23%)
3	167 (56%)
Not available	25 (8%)
Median follow-up from diagnostic (months)	41
Median follow-up from trabectedin line (months)	11
Median age, years (range)	51 (14–79)

With a median follow-up of 41 months from the diagnosis time, the median PFS with trabectedin was 3.4 months (95% confidential interval [CI] 2.9–4.0), and the median of OS measured from the trabectedin initiation was 12 months (95% CI 9.8–14.7). Among 268 (89%) patients who were evaluable for trabectedin response by RECIST v.1.1, eight (3%) patients had a complete response, 31 (10%) had a partial response, 91 (30%) had stable disease and 137 (46%) had progressive disease as the best response. The overall response rate (ORR) of this series for trabectedin was 13% (n = 39), while the clinical benefit (patients with response or stable disease) was 43% (n = 130). RECIST responses were seen in patients diagnosed with myxoid liposarcoma (n = 12), leiomyosarcoma (n = 10), dedifferentiated liposarcoma (n = 7), synovial sarcoma (n = 6), and other subtypes (n = 4).

#### Univariate analysis of clinical variables

In the univariate analysis of clinical factors, the diagnostic of L-sarcoma was correlated with a better PFS following trabectedin treatment [5.0 months (95% CI 3.4–6.5) vs 2.6 months (95% CI 2.0–3.2; p =  < 0.001] and better OS [18.8 months (95% CI 13.9–23.6) vs 6.1 months (95% CI 4.7–7.6); p =  < 0.001]. On the other hand, grade 3 tumors were associated with a worse PFS [3.0 months (95% CI 2.5–3.5) vs 5.1 months (95% CI 1.9–8.3); p =  < 0.001] and worse OS [8.5 months (95% CI 6.1–10.9) vs 18.3 months (95% CI 13.4–23.2); p =  < 0.001]. Other factors with a significant association with worse PFS were age above 51 years and metastatic disease at diagnosis, whereas age above the median was also significantly associated with worse OS measured from trabectedin treatment initiation (Supplementary Table S1).

#### RNA expression

The expression of selected *HMG* genes was heterogeneous among the 133 cases included in this analysis, with *HMGA2* (median log2 4.57; range − 0.46 to 14.30) and *HMGB2* (median log2 9.28; range 7.06–11.58) being the most underexpressed and overexpressed genes, respectively, among the five transcripts assessed. The median log2 expression of *HMGA1* was 7.46 (range 5.38–12.39), of *HMGB1,* was 9.12 (range 5.99–10.54), and of *HMGB3* was 5.55 (range − 0.46 to 8.16)—Supplementary Table S2. The expression of *HMGA1* expression positively correlated with *HMGA2* (p = 0.024) and *HMGB1* (p = 0.001) expression. Other significant correlations among the expression of HMG genes are shown in Supplementary Fig. S1.

#### Univariate analysis of RNA expression

**Fig. 1:**
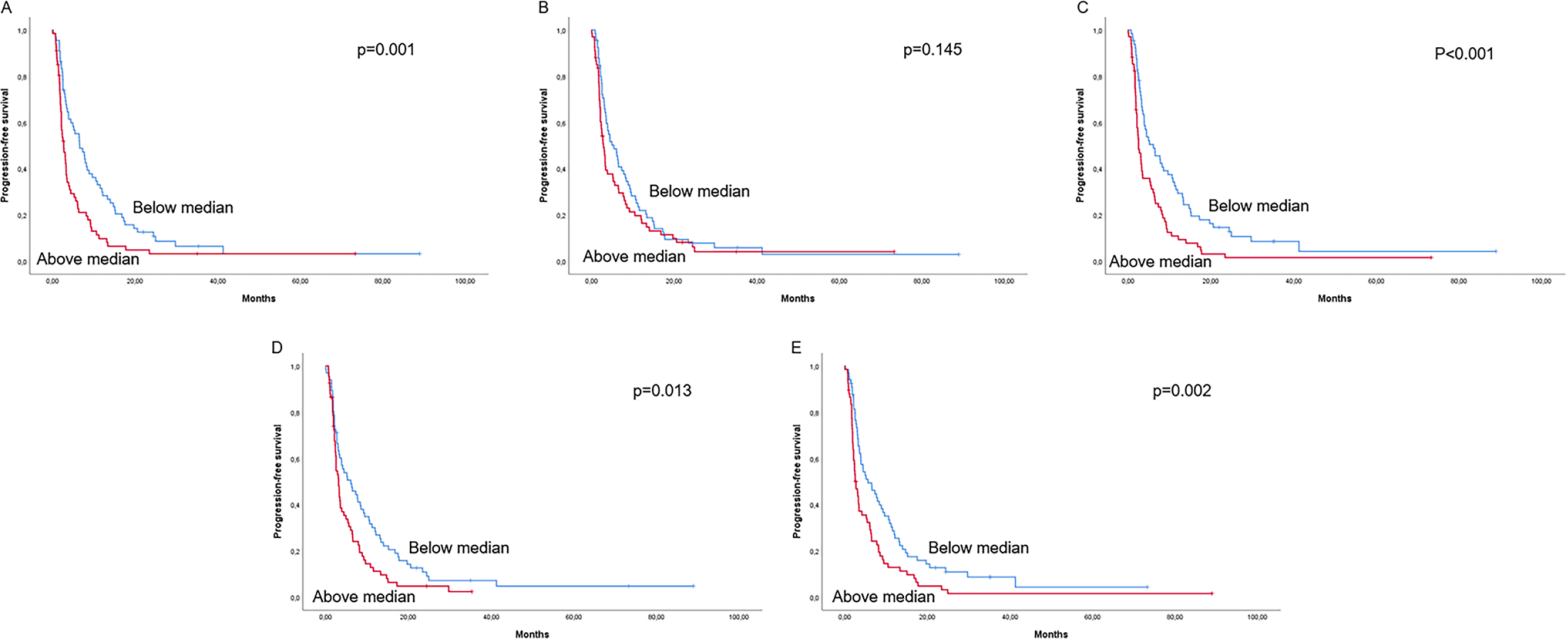
Univariate progression-free survival (PFS) analysis. **A** PFS according to *HMGA1* gene expression; **B** PFS according to *HMGA2* gene expression; **C** PFS according to *HMGB1* gene expression: **D** PFS according to *HMGB2* gene expression; and **E** PFS according to *HMGB3* gene expression. The groups were defined according to the median of gene expression: above (N = 66) and below (N = 67) the median. Log‐rank test statistical significance was defined at p ≤ 0.05

In the univariate analysis, the overexpression of *HMGA1*, *HMGB1*, *HMGB2,* and *HMGB3* was significantly associated with shorter PFS of trabectedin, while the expression of *HMGA2* did not show any statistically significant association (Supplementary Table S3 and Fig. 1). Regarding OS, measured from the time of trabectedin treatment initiation, the overexpression of *HMGA1*, *HMGB1,* and *HMGB3* were associated with worse OS, whereas *HMGA2* and *HMGB2* did not correlate with OS (Supplementary Table S3 and Supplementary Fig. S2). In the multivariate analysis, *HMGA1* [HR = 1.77 (95% CI 1.19–2.61), p = 0.004], *HMGB1* [HR = 1.59 (95% CI 1.07–2.37), p = 0.023] and *HMGB2* [HR = 1.74 (95% CI 1.19–2.57), p = 0.005] gene expression and diagnosis of L-sarcoma [HR = 0.61 (95% CI 0.41–0.89), p = 0.010] were the only variables independently associated with PFS (Supplementary Table S4).

Importantly, the gene expression of HMGs did not correlate significantly with the PFS of the previous line to the trabectedin line (Supplementary Table S5). Previous line included patients treated with doxorubicin-based schemes (n = 59), gemcitabine combinations (n = 45) and other drugs (n = 36).

#### Protein expression

The two genes with the most significant impact on trabectedin PFS in the univariate analysis (*HMGA1* and *HMGB1*), were selected for protein expression assessment. A total of 301 tumor samples were used for this analysis. The extension of HMGA1 expression was high in 48 out of 301 (16%), low in 224 out of 301 (74%), and non-evaluable in 29 out of 301 (10%) patients. The extension of HMGB1 expression was high in 204 out of 301 (68%), low in 68 out of 301 (22%), and non-evaluable in 29 out of 301 (10%) patients. The intensity of HMGA1 immunostaining was strong in 66 out of 301 (21%), weak-negative in 206 out of 301 (69%), and non-evaluable in 29 out of 301 (10%) patients. The intensity of HMGB1 immunostaining was strong in 189 out of 301 (63%), weak-negative in 83 out of 301 (27%), and non-evaluable in 29 out of 301 (10%) patients (Table 2). An example of positive and negative HMGA1 immunostaining can be found in Supplementary Fig. S3. Protein expression was non-evaluable in 10% of the samples due to tissue limitations, which made the core cylinders detach from the TMAs.

**Table 2:** HMGA1 and HMGB1 protein expression

	HMGA1	HMGB1
Expression		
Low	224 (74%)	68 (22%)
0–4%	177 (59%)	36 (11%)
5–24%	28 (9%)	14 (5%)
26–49%	19 (6%)	18 (6%)
High	48 (16%)	204 (68%)
50–100%	48 (16%)	204 (68%)
Non-evaluable	29 (10%)	29 (10%)
Intensity		
Weak-negative	206 (69%)	83 (27%)
Negative	177 (59%)	36 (11%)
Weak	29 (10%)	47 (16%)
Strong	66 (21%)	189 (63%)
Strong	66 (21%)	189 (63%)
Non-evaluable	29 (10%)	29 (10%)

#### Univariate analysis of protein expression

**Fig. 2:**
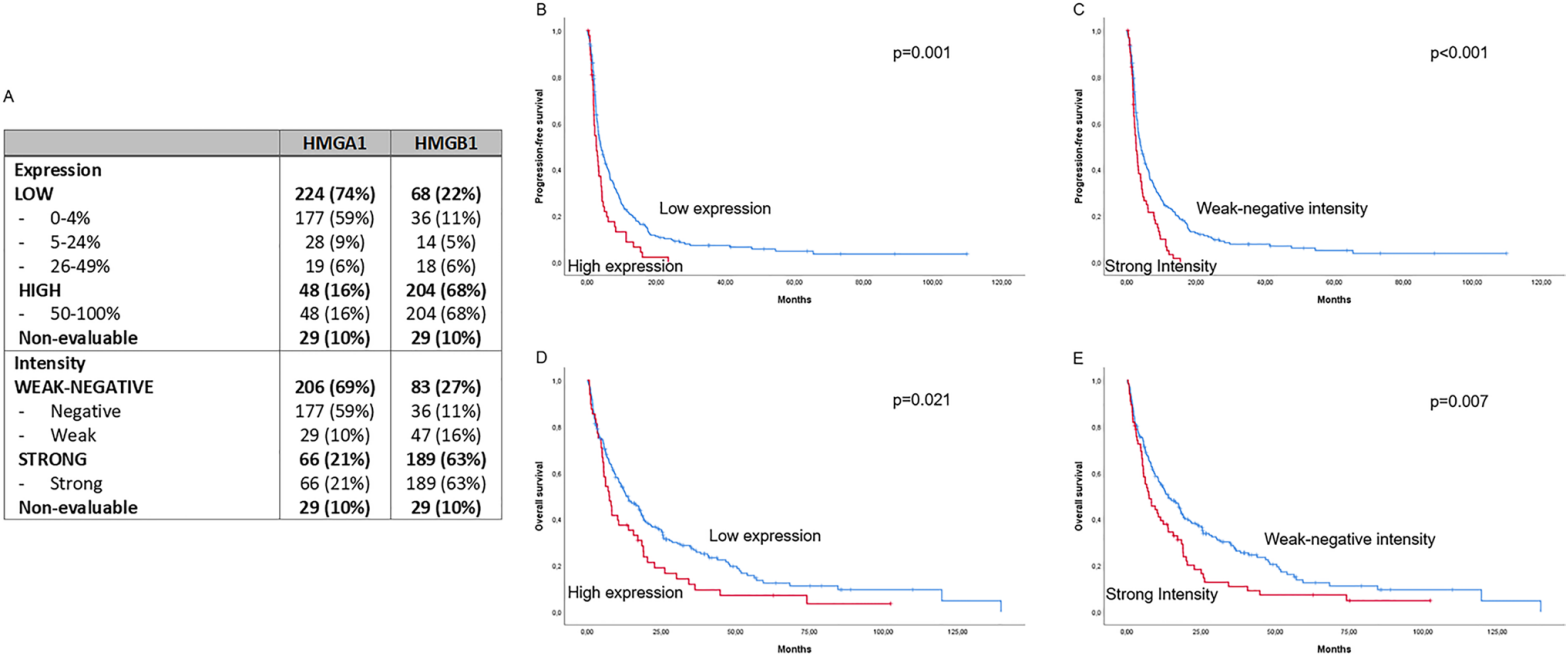
Prognostic value of HMGA1 protein levels. **A** Distribution of HMGA1 and HMGB1 protein expression and intensity; **B** progression-free survival (PFS) according to HMGA1 protein expression (low expression, N = 224 vs. high expression, N = 48); **C** PFS according to HMGA1 immunostaining intensity (weak-negative, N = 206 vs. strong, N = 66); **D** Overall survival (OS) according to HMGA1 protein expression (low expression, N = 224 vs. high expression, N = 48); **E** OS according to HMGA1 immunostaining intensity (weak-negative, N = 206 vs. strong, N = 66)

In the univariate analysis, higher HMGA1 protein expression significantly correlated with a worse PFS [2.6 months (95% CI 1.6–3.6) vs. 3.9 months (95% CI 2.8–5.0), p = 0.001], and worse OS [7.3 months (95% CI 4.9–9.7) vs. 13.1 months (95% CI 9.1–17.1), p = 0.021]—Fig. 2. HMGB1 protein expression did not correlate with either PFS or OS (Supplementary Table S6).

Similarly, strong HMGA1 intensity was significantly associated with worse PFS [2.6 months (95% CI 1.9–3.4) vs. 4.0 months (95% CI 3.0–5.0), p < 0.001] and worse OS [7.4 months (95% CI 3.7–11.2) vs. 13.9 months (95% CI 9.7–18.1), p = 0.007]—Fig. 2. HMGB1 protein intensity failed to correlate with either PFS or OS (Supplementary Table S6).

Applying a histoscore to our protein results that consider both the expression and the intensity of the immunostaining, we observed that cases with a histoscore of 4 to 6 (N = 51), have significantly worse PFS [2.5 months (95% CI 1.8–3.3) vs. 4.1 months (95% CI 3.1–5.1), p < 0.001], compared to the cases with a histoscore for HMGA1 protein levels lower than 4 (N = 221). Patients with a histoscore of 4 to 6 have also significantly worse OS [7.4 months (95% CI 4.1–10.8) vs. 13.8 months (95% CI 9.6–18.0), p = 0.012].

A sub-analysis was performed for HMGA1 according to the histological subtype. In L-sarcomas, HMGA1 strong intensity was significantly associated with worse PFS [3.2 months (95% CI 2.0–4.4) vs. 5.6 months (95% CI 4.1–7.1), p = 0.008] and worse OS [10.5 months (95% CI 0.8–20.2) vs. 21.8 months (95% CI 16.0–27.6), p = 0.017]. Nonetheless, the extension of the expression of HMGA1 did not correlate significantly with survival in L-sarcomas (Supplementary Table S7). In non-L-sarcomas, neither expression nor the intensity of HMGA1 immunostaining correlated significantly with PFS or OS (Supplementary Table S8). Of note, the prognostic value of HMGA1 intensity and expression for trabectedin PFS was significantly marked in leiomyosarcoma cases (Supplementary Table S9).

### Preclinical research

#### HMGA1 expression in soft-tissue sarcoma preclinical models

**Fig. 3:**
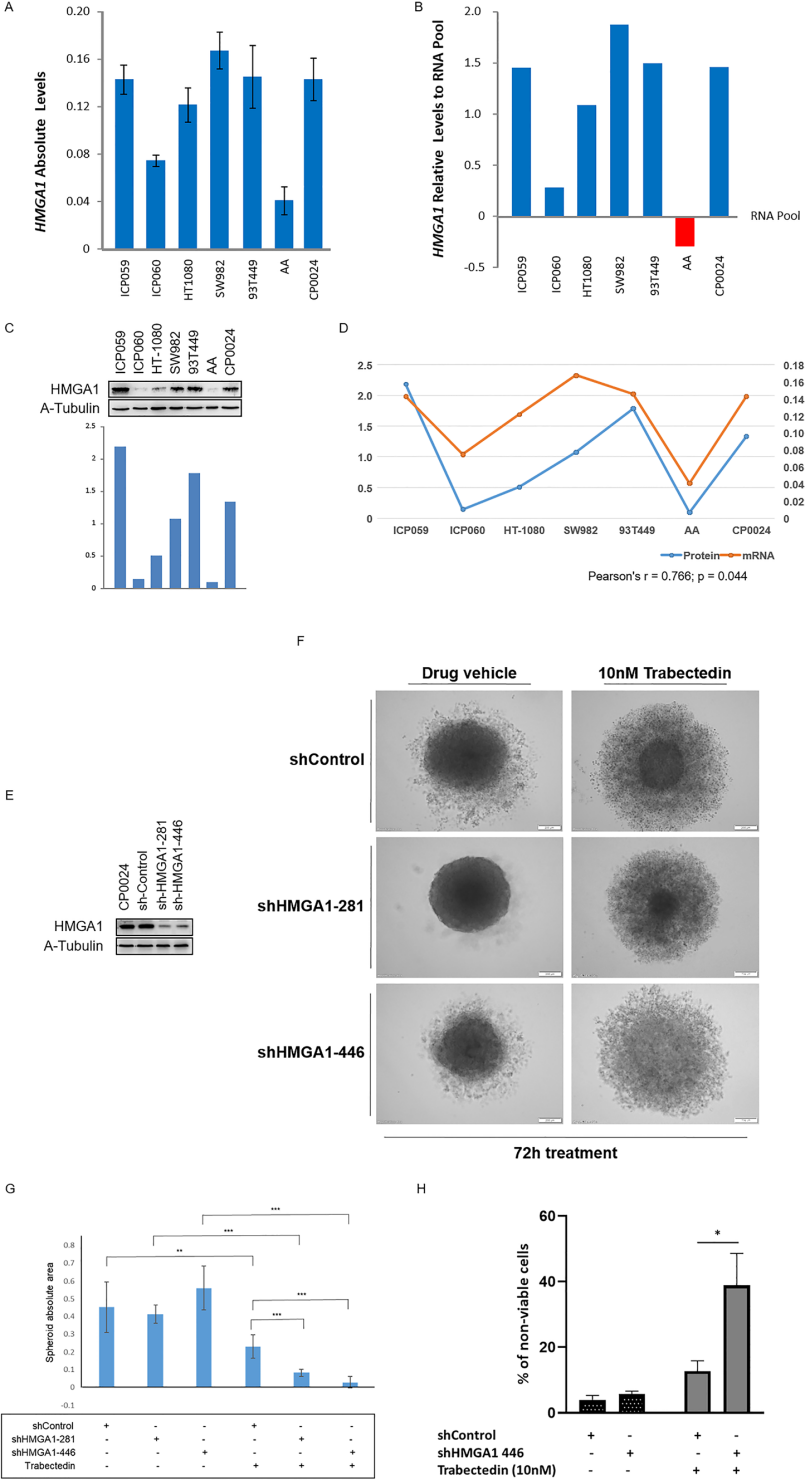
HMGA1 expression levels and effect of HMGA1 downregulation in trabectedin sensitivity in in vitro models of soft-tissue sarcoma (STS). **A** *HMGA1* gene expression levels in STS cell lines; **B** relative expression of *HMGA1* mRNA levels in STS cell lines to an external human RNA pool. **C** HMGA1 protein expression levels in STS cell lines; **D** correlation between mRNA and protein expression levels in the panel of STS cell lines used. Pearson’s r-test statistical significance was defined at p ≤ 0.05; **E** depletion of HMGA1 in CP0024 cells, using two different shRNAs. HMGA1 was knocked down and the level of HMGA1 protein was determined by western blot. **F** Effect of HMGA1 knockdown on the formation of 3D spheroids of CP0024 cells treated with 10 nM trabectedin for 72 h. **G** Effect of HMGA1 knockdown in the area of the CP0024 3D spheroid treated with 10 nM trabectedin for 72 h. **H** Percentage of non-viable cells (apoptotic, early apoptotic, and necrotic cells) measured by flow cytometry after 24 h of treatment with 10 nM trabectedin (n = 3). CP0024 cells were transduced with a shHMGA1 or with a non-targetting shControl, before treatment. Student t-test statistical significance was defined at p ≤ 0.05 (*), p ≤ 0.005 (**) or p ≤ 0.0005 (***). Spheroid areas were determined with ImageJ

The expression levels of HMGA1 were determined by mRNA and protein in a panel of 7 STS cell lines, to compare the expression of leiomyosarcoma cell lines with other STS histological subtypes. *HMGA1* gene expression was high in CP0024 leiomyosarcoma 2D cell cultures, but similar to angiosarcoma (ICP059), sarcoma (SW982), and liposarcoma (93T449) cell lines. The AA leiomyosarcoma cell line showed the lowest expression of *HMGA1* (Fig. 3A and B) in our experimental conditions. Analogous results were obtained at protein level (Fig. 3C). A significant and positive correlation between HMGA1 mRNA and protein expression levels was observed in our experiments (Fig. 3D).

#### HMGA1-dependent trabectedin resistance

Trabectedin drug resistance was tested in 3D spheroids and 2D monocultures after transducing the CP0024 cell line (leiomyosarcoma cell line with HMGA1 highest expression levels) with lentiviral particles containing shRNAs against HMGA1 (shHMGA1) and a control non-targeting shRNA (shControl). The decrease in HMGA1 protein expression was similar between both the shRNAs tested (Fig. 3E). At 72 h after treating our 3D cultures with 10 nM trabectedin, HMGA1 knockdown affected the capacity of tumor spheroids to grow: shHMGA1 spheroids showed a significant decrease in their compacted area, compared to the shControl (Fig. 3F): 0.230 mm^2^ ± 0.067mm2 (shControl) vs. 0.083 mm^2^ ± 0.020 mm^2^ (shHMGA1-281), p < 0.001 and 0.230 mm^2^ ± 0.067 mm^2^ (shControl) vs. 0.029 mm^2^ ± 0.033 mm^2^ (shHMGA1-446), p < 0.001 (Fig. 3G). In 2D monocultures, the knockdown of HMGA1, increased significantly the percentage of non-viable cells (apoptotic, early apoptotic, and necrotic cells) after trabectedin treatment, compared to the control cells: 12.72% ± 3.18% vs. 38.87% ± 9.78%, p = 0.012).

#### Effect of HMGA1 knockdown on gene expression

**Fig. 4:**
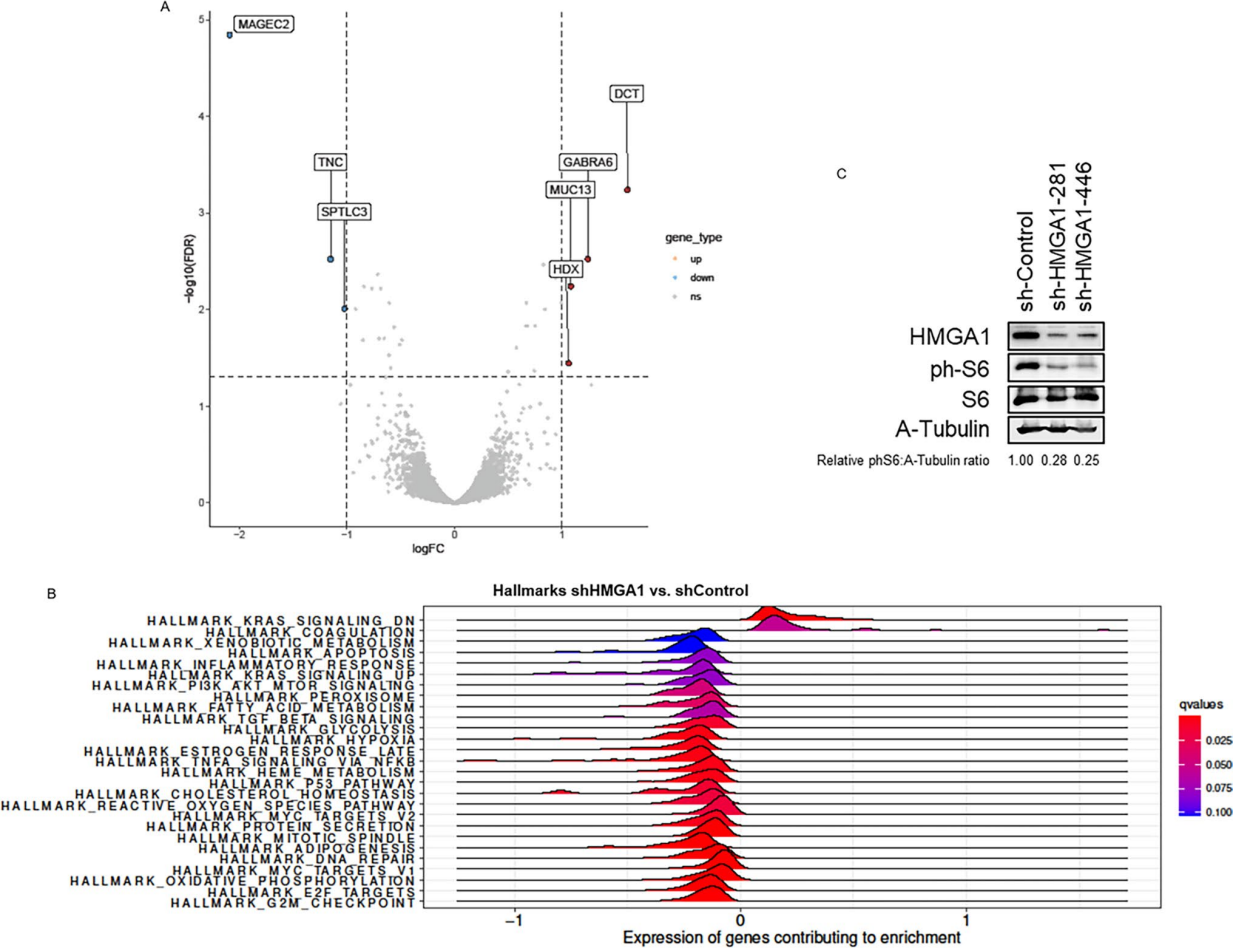
Effect of HGMA1 silencing in gene expression. **A** Volcano plot with the top significant genes (adjusted p-value < 0.05 and logFC < − 1 or > 1), comparing shHMGA1 vs. shControl; **B** enrichment analysis in Hallmark gene sets of the effect of HMGA1 knockdown and **C** effect of HMGA1 silencing in mTOR cell signaling pathway target phospho-S6, by western blot, in CP0024 leiomyosarcoma cells. Protein expression levels were quantified with Image J

An Affymetrix Clarion S Human array was performed to identify the genes and the cellular pathways affected by the downregulation of HMGA1 levels in the CP0024 cells and that could justify the increased sensitivity to trabectedin treatment. A total of 33 genes were significantly and differently expressed, by multiple comparisons (adjusted p-value < 0.05), when analyzing and comparing mRNAs extracted from cell cultures transduced with shHMGA1 (data from both shRNAs were analyzed together) and with the non-targeting shControl (Supplementary Table S10). The most overexpressed and underexpressed genes in CP0024 leiomyosarcoma cell cultures transduced with shHMGA1 were *DCT* (logFC = 1.607; adjusted p-value < 0.001), and *MAGEC2* (logFC = − 2.091; adjusted p-value < 0.001), respectively (Fig. 4A). Functional enrichment analysis identified 23 *Hallmarks* gene sets significantly (adjusted p-value < 0.05) altered after the knockdown of HMGA1 (Fig. 4B): 2 Hallmark gene sets enriched in shHMGA1 cells (positive NES value) and 21 Hallmark gene sets enriched in shControl cells (negative NES value). Among these *Hallmarks,* several pathways related to proliferation (e.g. HALLMARK_E2F_TARGETS; NES: − 2.381; adjusted p-value < 0.001), cell cycle (e.g. HALLMARK_G2M_CHECKPOINT; NES: − 2.466; adjusted p-value < 0.001) and DNA damage repair (DDR; e.g. HALLMARK_DNA_REPAIR; NES: − 1.848; adjusted p-value < 0.001) and CSC maintenance (e.g. HALLMARK_MYC_TARGETS_V1; NES: − 1.884; adjusted p-value < 0.001) were downregulated in shHMGA1 cell cultures, compared to shControl-transduced cells. Noteworthy, another *Hallmark* related to CSC maintenance significantly altered in shHMGA1 cells was PI3K/AKT/mTOR cell signaling pathway (HALLMARK_PI3K_AKT_MTOR_SIGNALING; NES: − 1.366; adjusted p-value = 0.048). By western blot, we observed that HMGA1 silencing induces a decrease in the protein expression levels of phospho-S6, a target of phosphorylation by the mTOR cell signaling pathway (Fig. 4C). In CP0024 leiomyosarcoma cells, HMGA1 silencing decreases the levels of phospho-S6, ranging from 17% (shHMGA1-281) to 34% (shHMGA1-446).

#### Combination of mTOR inhibitor and trabectedin: a proof of concept

**Fig. 5:**
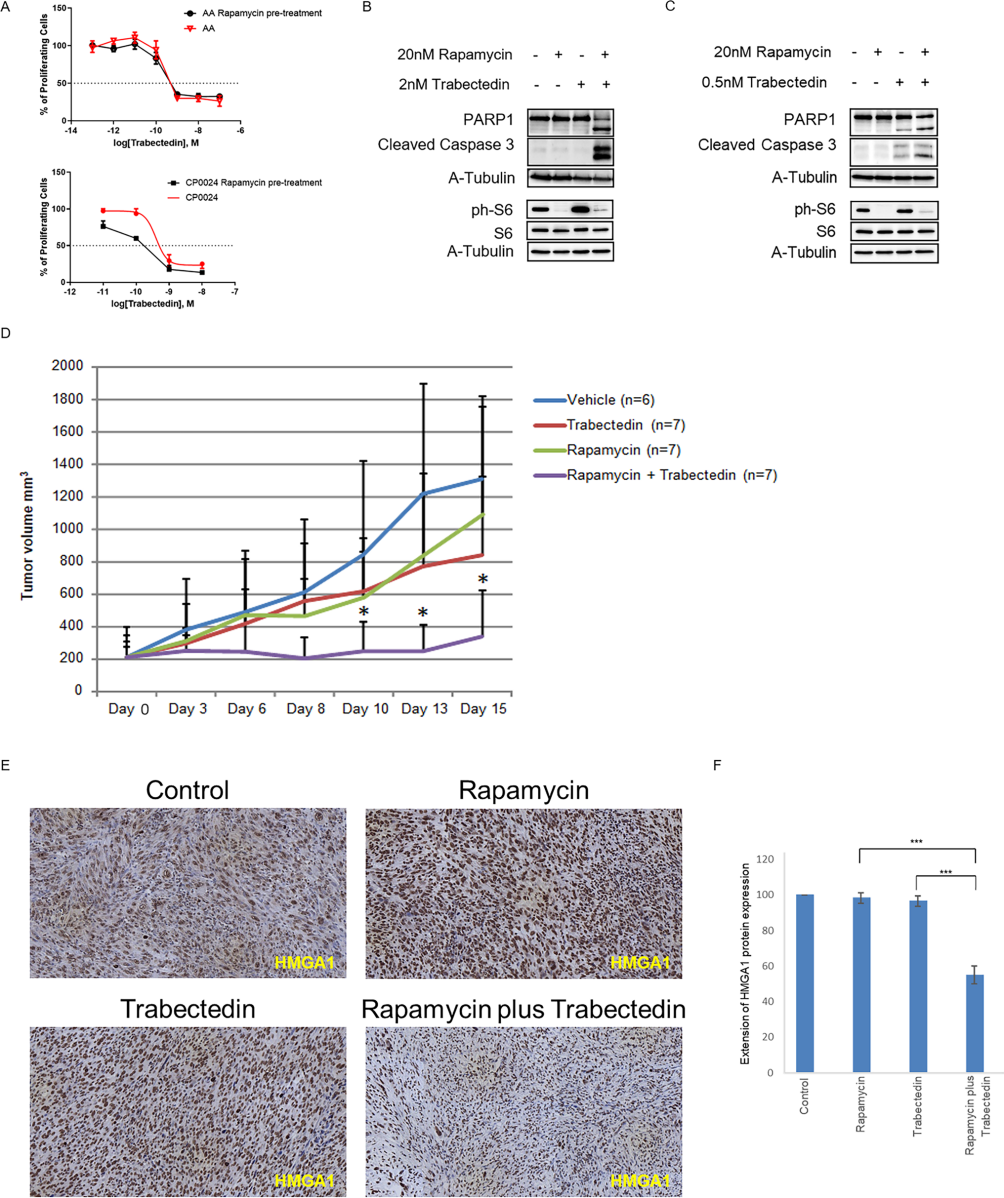
Efficacy of rapamycin plus trabectedin combination in soft-tissue sarcoma preclinical models. **A** Percentage of proliferating cells in the CP0024 and AA cell lines treated with increased concentrations of trabectedin (10E-11 to 10E-8) for 72 h (red curve) and in CP0024 and AA cells pre-treated with 20 nM rapamycin for 2 h, before trabectedin treatment (10E-11 to 10E-8) for 72 h (black curve). Three biological replicates with three technical replicates were performed. **B** Protein expression, by western blot, of apoptotic makers (cleaved PARP1 and cleave Caspase 3) after 24 h of treatment with 20 nM rapamycin and 2 nM trabectedin, as single agents or in combination in CP0024 cell line. **C** Protein expression, by western blot, of apoptotic makers (cleaved PARP1 and cleave Caspase 3) after 24 h of treatment with 20 nM rapamycin and 0.5 nM trabectedin, as single agents or in combination in AA cell line. **D** Tumor volume (mm^3^) of mice treated with intraperitoneal rapamycin (0.5 mg/kg) two times a week (*q7dx2*) and intravenous trabectedin (0.15 mg/kg) once a week (*q7dx1*), as single agents or in combination. DMSO was used as the control vehicle. **E** HMGA1 protein expression was analyzed by immunohistochemistry in the tumors collected from mice (200x). **F** Extension of HMGA1 protein expression levels quantification. Student t-test statistical significance was defined at p ≤ 0.05 (*), p ≤ 0.005 (**) or p ≤ 0.0005 (***)

HMGA1 inhibitors are currently unavailable for preclinical or clinical research. Thus, we hypothesize that inhibiting a downstream pathway governed by HMGA1 could increase the sensitivity to trabectedin. In this case, the combination of rapamycin (i.e. mTOR inhibitor) plus trabectedin was performed as a proof of concept in leiomyosarcoma in vitro models. First, we analyzed the effect of rapamycin pre-treatment (2 h before trabectedin treatment; rapamycin was washed out before trabectedin treatment) in the sensitivity of both leiomyosarcoma cell lines CP0024 (higher levels of HMGA1) and AA (lower levels of HMGA1). By calculating the IC_50_ values of trabectedin, we observed a decrease in the IC_50_ values in both CP0024 [399 pM (95% CI 120–1393) to 176 pM (95% CI 109–281)] and AA [218 pM (95% CI 64–738) to 178 pM (95% CI 94–335)] cell lines (Fig. 5A) when our cell lines were pre-treated with rapamycin. However, the decrease observed for each cell line does not seem to reach statistical significance, since the 95% CIs of trabectedin and combination IC_50_ overlap. Of note, the decrease in the trabectedin IC_50_ was markedly greater in cells with higher expression of HMGA1 [CP0024 (56%)], compared to those with lower expression of HMGA1 [AA (18%)]. The combination index value at effective dose at 50% (ED) for the combination of rapamycin and trabectedin, calculated with CalcuSyn software was 0.129 and 0.027, for CP0024 and AA, respectively, which means strong synergism [34]. To understand if the potentiated effect of trabectedin was due to increased cell death by apoptosis we analyzed the expression of apoptotic markers by western blot after exposing CP0024 cells to 20 nM rapamycin and/or 2 nM trabectedin. By western blot, we observed an increase in cleaved Caspase 3, as well as in cleaved PARP1 with the combination after only 24 h of treatment. In these experimental conditions, neither rapamycin nor trabectedin as single agents induced the cleavage of apoptotic markers (Fig. 5B). By western blot, we also analyzed the protein expression levels of phospho-S6, a target of phosphorylation by the mTOR pathway. As expected, the levels of phospho-S6 were downregulated after rapamycin treatment. Of note, the levels of phospho-S6 increased in CP0024 leiomyosarcoma cells after trabectedin treatment; however, rapamycin treatment was shown to reverse this increase (Fig. 5B). On the other hand, the AA cell line was treated with 20 nM rapamycin and/or 0.5 nM trabectedin, since this cell line was more sensitive to this drug. By western blot, we observed an increase in cleaved Caspase 3, as well as in cleaved PARP1 with the combination after only 24 h of treatment. In these experimental conditions, trabectedin as a single agent induced the cleavage of apoptotic markers PARP1 and Caspase 3. The increase in the levels of phospho-S6 was inferior compared to the CP0024 cell lines; rapamycin treatment decreased the levels of phospho-S6 in the AA cell line, in a similar manner as observed for the CP0024 cell line (Fig. 5C).

Moreover, with the final aim to validate the activity of this combination, we completed an in vivo study, using an immunocompetent fibrosarcoma-like model induced by the intramuscular injection of 3-MC. In our in vivo experiments, the combination of rapamycin plus trabectedin induced a significant stabilization of tumor growth, for the duration of the treatment (5 days), compared to trabectedin in monotherapy (339.88 mm^3^ ± 284.91 mm^3^ vs. 839.03 mm^3^ ± 487.39 mm^3^, p = 0.037) and rapamycin as a single agent (339.88 mm^3^ ± 284.91 mm^3^ vs. 1088.51 mm^3^ ± 665.06 mm^3^, p = 0.020)—Fig. 5D. During the experiment, we had to sacrifice one mouse in the control group treated with the vehicle on day 10 and one mouse treated with rapamycin on day 8 of treatment, probably due to the aggressiveness of the 3-MC tumors. In terms of body weight, only two mice had a decrease in body weight greater than 10% in the combination group (Supplementary Fig. S4); however, without clinical malaise. The extension of HMGA1 protein expression levels by immunohistochemistry was analyzed by an expert pathologist and these levels were downregulated in the combination group, compared to both rapamycin and trabectedin as single agents, in the tumors collected from mice (Fig. 5E). The levels of phospho-S6 decreased in rapamycin-treated groups (Supplementary Fig. S5).

## Discussion

The prognostic value of HMGA1 mRNA and protein expression has been shown for trabectedin PFS and OS in a series of 301 patients with advanced STS. Among the five HMG genes selected and evaluated in our study, the overexpression of *HMGA1*, *HMGB1*, *HMGB2,* and *HMGB3* were significantly associated with worse PFS for trabectedin, whereas *HMGA2* expression did not correlate with survival. In the multivariate analysis, high expression of *HMGA1*, *HMGB1,* or *HMGB2* were independently associated with worse PFS. Nevertheless, HMGA1 was the factor that sustained the most significant associations with trabectedin outcome, mostly in patients diagnosed with L-sarcomas and in particular in leiomyosarcoma. These results are in line with data previously published, in which it was shown that HMGA1 regulated several pro-tumoral processes in dedifferentiated and myxoid liposarcoma, including resistance to trabectedin treatment [26]. In fact, in this study, it was shown that HMGA1 depletion in combination with trabectedin treatment had an additive effect on myxoid liposarcoma cell death, which demonstrated that this epigenetic transcriptional factor plays an important role in the mechanisms of resistance to trabectedin [26]. In our preclinical experiments, we observed a similar effect, with HMGA1 knockdown, increasing the percentage of cell death, measured by flow cytometry. Nonetheless, an association between high expression of HMGA1 and increased activity of trabectedin on thyroid and colon carcinoma cells has been also reported [30]. In these preclinical experiments, trabectedin seems to dislocate HMGA1 from the HMGA1-responsive promoters (e.g. ATM), impairing their transcriptional activity. As a consequence, DNA damage repair mechanisms could be affected, increasing the accumulation of DNA damage after trabectedin treatment [30]. Likewise, it has also been reported that trabectedin seems to downregulate the expression of HMGA1-targetted genes in anaplastic thyroid carcinoma [35]. Although tumor histology may justify the different results observed between our study and these publications, we need to take also into account the experimental context (human samples vs. cell lines and immunosuppressed mice model) in the case of D’Angelo and colleagues work [30], and the role of HMGA1 in modulating inflammation and milieu-associated CSCs [36], which may ultimately affect the sensitivity to drug treatment.

Additionally, it has also been reported that HMGA1 regulated drug resistance in liposarcomas, through a mechanism involving E2F1 [26]. The regulation of E2F1 and its target genes by HMGA1 had been previously published [36, 37] and these results are in line with our enrichment analysis data, in which we observed a downregulation in a set of genes related to the *Hallmark targets of E2F*, in cells silenced for HMGA1. Curiously, trabectedin treatment seems to upregulate E2F1 in multiple myeloma [38], which supports the hypothesis that the HMGA1/E2F1 axis could be activated as a mechanism of resistance after trabectedin. It is also plausible that the activation of this axis induced the expression of cell cycle proliferative factors, such as cyclins [39, 40], through the initial phases of the cell cycle, leading to a quick and sustained accumulation of cells in G2/M [41, 42]. This proliferative signal and the G2/M cell cycle arrest are key for the radiosensitizer role of trabectedin [42–44] and it could justify the great results obtained with the combination of trabectedin and low-dose radiotherapy in patients with metastatic STS [42, 45, 46]. In the non-randomized phase 1/2 clinical trial in metastatic STS the ORR was 72% and 60% in local and central assessments [42]. Future translational research associated with this trial should determine the prognostic and/or predictive value of HMGA1 in these patients treated with trabectedin and radiation therapy. Additionally, our enrichment analysis data showed that several other sets of genes were affected by HMGA1 silencing, some of them regulating pathways involving NFKB, MYC, or mTOR, which have been previously described as participating in the maintenance of CSCs-mediated drug resistance [47–49]. While the NF-κB pathway has already been described as being associated with trabectedin resistance in myxoid liposarcoma [26], we decided to explore the inhibition of the mTOR pathway in combination with trabectedin, since to our knowledge, specific HMGA1 inhibitors are not currently available for research. Although there is no evidence in sarcomas, it has been described in skin cancer cells that HMGA1 depletion disturbs the activity of the mTOR pathway [50], which is in line with our observations that HMGA1 silencing in leiomyosarcoma cells affects the mTOR pathway. The PI3K/AKT/mTOR pathway is aberrantly activated in many types of cancers, including sarcomas [51], and this pathway plays a pivotal role in the development of leiomyosarcomas [52]. In line with these observations, it has been recently reported that the HMGA2/IGF2BP/IGF2/IGF1R/AKT/mTOR axis was typically upregulated in capicua-double homeobox 4 (CIC-DUX4)–rearranged sarcomas and renders these tumors sensitive to the combination of trabectedin with PI3K/mTOR inhibitors [53]. While we did not evaluate the effect of HMGA2 knockdown in our preclinical studies, both HMGA1 and HGMA2 may be linked to the upregulation of the PI3K/AKT/mTOR pathway. In leiomyosarcoma cells, we showed that the combination of trabectedin and rapamycin is synergistic and that pre-treatment with rapamycin may sensitize cells for trabectedin treatment. This synergy was associated with an increase in the expression of apoptotic markers. Likewise, this combination was shown to be active in a highly aggressive immunocompetent model of sarcoma, stabilizing tumor growth during the duration of the experiment. This stabilization was associated with decreased expression of HMGA1 protein levels in the tumor, suggesting that the combined treatment was capable to downregulate HMGA1 expression and therefore the resistance to trabectedin. In this model, neither trabectedin nor rapamycin in monotherapy were able to arrest tumor growth. It is worth mentioning that similar results were previously reported in clear cell ovary carcinoma for the combination of trabectedin with mTOR inhibitors [54]. In this histological subtype, mTOR inhibition with everolimus enhanced the activity of trabectedin in both in vitro and in vivo models, while preventing the activation of mechanisms of resistance, dependent on mTOR pathway triggering [54]. The combination of everolimus with the marine-derived analog of trabectedin, and lurbinectedin showed similar results in the same histology [55], confirming the added value of combining mTOR inhibitors with trabectedin. Moreover, an increased activation of the mTOR cell signaling pathway was described in trabectedin-resistant clear cell carcinoma cell lines, while trabectedin treatment induced a prolonged stimulation of mTOR in the same model. The inhibition of the mTOR pathway prevented clear cell carcinoma cells from acquiring resistance to trabectedin [54]. In our experimental conditions, we observed a similar effect; trabectedin treatment increases the phosphorylation of the ribosomal protein S6, an effect that is avoided by treating leiomyosarcoma cells with mTOR inhibitor rapamycin. Additionally, our group reported a clinical case of a uterine leiomyosarcoma patient who experienced stable disease for 30 months when treated with trabectedin; however, the most remarkable fact is that this patient, before starting trabectedin treatment, was treated within a clinical trial with the mTOR inhibitor ridaforolimus until progression [56]. Thus, it seems that the patient could have benefitted from mTOR inhibition, by downregulating potential mechanisms of resistance to trabectedin, which is in line with our preclinical observations.

Besides HMGA1, our results also showed that other HMGs, including HMGB1, HMGB2, and HMGB3 might have a prognostic and/or predictive value for trabectedin in STS patients. We were not able to validate the prognostic value of *HMGB1* in terms of protein expression, whereas validation by immunohistochemistry of the results obtained for *HMGB2* and *HMGB3* was not performed due to budget restrictions. Concerning HMGB1, it has been reported that the high expression of this gene correlated significantly with worse PFS for the combination of trabectedin with low-dose radiotherapy in advanced STS [42]. Moreover, it is known that this biomarker of immunogenic cell death is released in response to lurbinectedin treatment, an effect that can boost the efficacy of immunotherapy-based regimens, as reported also in STS with the combination of chemotherapy and immune checkpoint inhibitors [57, 58]. While we could validate, at least in terms of gene expression, the prognostic value of HMGB1 for trabectedin PFS in advanced STS, we did not perform dynamic studies to analyze the effect of trabectedin treatment in the levels of HMGB1 protein expression. Thus, these observations should be tested in STS preclinical models to understand whether trabectedin treatment may induce immunogenic cell death, attract cytotoxic T-cells, inflame the sarcoma microenvironment, and potentiate the activity of immunotherapy. To the best of our knowledge, this is the first time that the expression of HMGB2 or HMGB3 has been associated with the efficacy of trabectedin. Future investigations should identify the mechanisms governed by these two epigenetic transcription factors that could trigger the resistance to trabectedin treatment.

Limitations of this study include the lack of protein validation for HMGB2 and HMGB3 and the fact that we should have tested an additional antibody for validating the prognostic value of HMGB1. Likewise, we should have evaluated the phospho-S6 protein expression in our series of cases and correlated it with the expression of HMGA1 and clinical outcome; however, due to budget limitations, we could not cover these analyses. Moreover, since some histologies were underrepresented in our series of cases, we cannot exclude that HMGA1 overexpression may have an impact on the efficacy of trabectedin and patient survival in other sarcoma subtypes, besides leiomyosarcomas. Another limitation is the lack of HMGA1 inhibitors for preclinical research. Thus, it was not possible to test the pharmacologic effect of inhibiting HMGA1 in the in vitro and in vivo efficacy of trabectedin. In silico molecular dynamics studies should be performed to identify potential molecules that could bind and inhibit HMGA1, to be tested in preclinical models of STS, in particular leiomyosarcoma.

Future preclinical studies should also address the potential role of DNA topoisomerase I/II poisons (i.e. irinotecan and doxorubicin, respectively) in the epigenetic regulation of HMGA1 in the enhanceosome. The combination of low doses of irinotecan or doxorubicin with trabectedin has shown to be active in prospective clinical trials in relapsed/refractory Ewing sarcoma [59] and leiomyosarcoma [60, 61], respectively, and this significant increase in activity could be related to epigenetic modulation of enhanceosome related complexes, such as the SWI/SNF chromatin remodelers [62]. Finally, we did not consider in our preclinical experiments, the potential impact of the combination of trabectedin and mTOR inhibitors in the sarcoma immune microenvironment. Trabectedin has been reported to deplete tumor-associated macrophages [63] in preclinical experiments [64, 65] and correlative studies associated with prospective clinical trials [66]. Future in vitro studies with multi-cultures of tumor and immune cells, as well as with multiplexed proteomics immunophenotyping, using the tumors collected from mice treated with trabectedin plus rapamycin should be performed to evaluate the effect of this combination in the immune milieu.

Overall, this translational and preclinical study identified HMGA1 as an independent prognostic factor associated with the lack of efficacy of trabectedin treatment in advanced STS. HMGA1 silencing increases the efficacy of trabectedin, through mechanisms that partially rely on the downregulation of the mTOR cell-signaling pathway. The combination of mTOR inhibitor with trabectedin was shown to be active in preclinical models of sarcoma, supporting the rationale for future clinical trials in STS.

### Supplementary Information

Below is the link to the electronic supplementary material.

Supplementary file1 (TIF 712 KB)

Supplementary file2 (TIF 1189 KB)

Supplementary file3 (TIF 4527 KB)

Supplementary file4 (TIF 1522 KB)

Supplementary file5 (TIF 6334 KB)

Supplementary file6 (DOCX 18 KB)

Supplementary file7 (DOCX 12 KB)

Supplementary file8 (DOCX 14 KB)

Supplementary file9 (DOCX 12 KB)

Supplementary file10 (DOCX 14 KB)

Supplementary file11 (DOCX 14 KB)

Supplementary file12 (DOCX 14 KB)

Supplementary file13 (DOCX 14 KB)

Supplementary file14 (DOCX 14 KB)

Supplementary file15 (DOCX 13 KB)

## Data Availability

Datasets containing HMG mRNA levels or from microarrays of HMGA1-knockdown cell lines are available from the corresponding author on reasonable request.
