# Supplementary figures and images for: HMGA1 regulates trabectedin sensitivity in advanced soft-tissue sarcoma (STS): A Spanish Group for Research on Sarcomas (GEIS) study
Source: Cell Mol Life Sci. 2024 May 17;81(1):219. doi: 10.1007/s00018-024-05250-y (PMC11101398; doi:10.1007/s00018-024-05250-y)

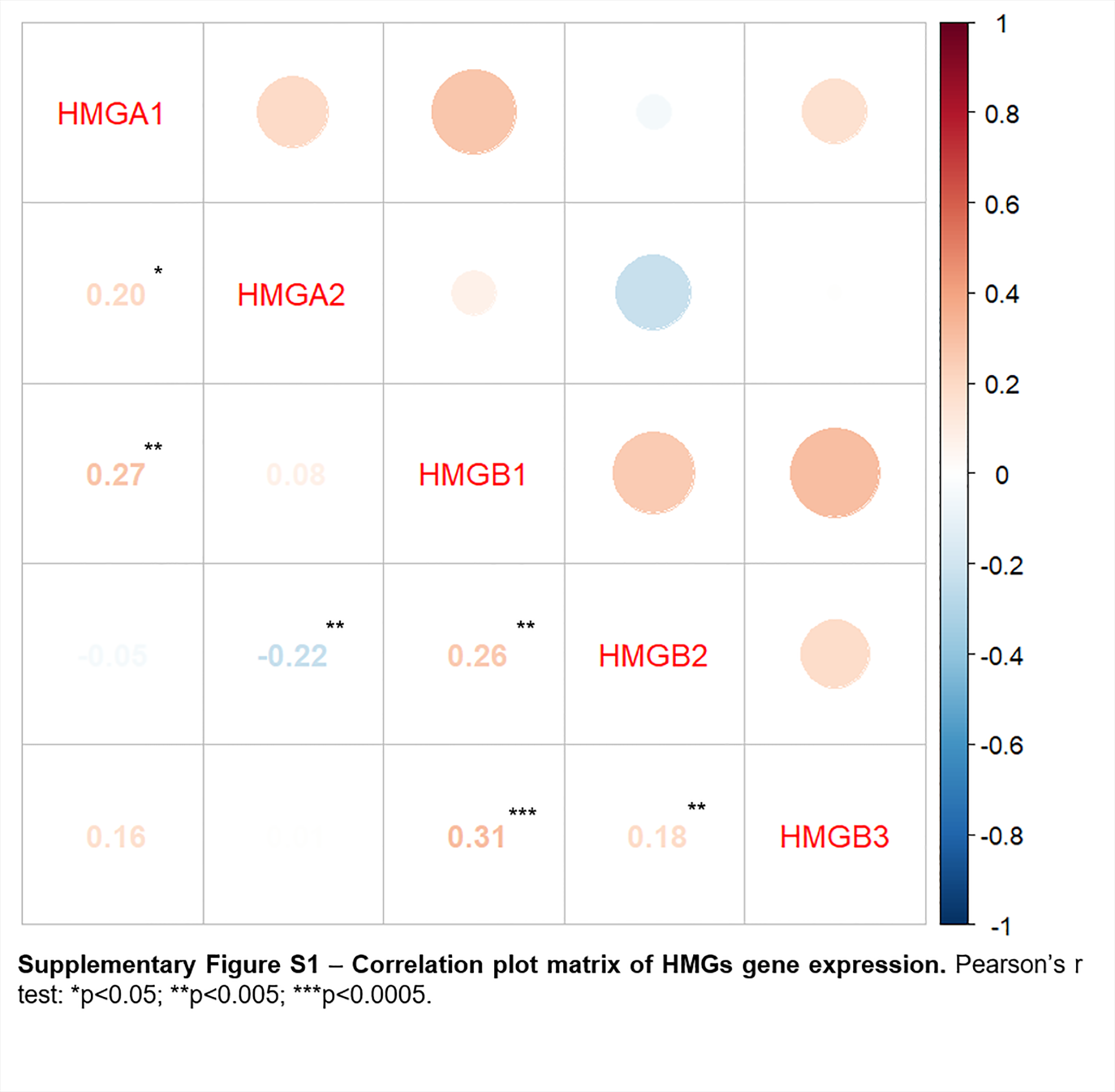

Supplement: Supplementary file 1 — Supplementary file1 (TIF 712 KB) [file 18_2024_5250_MOESM1_ESM.tif]

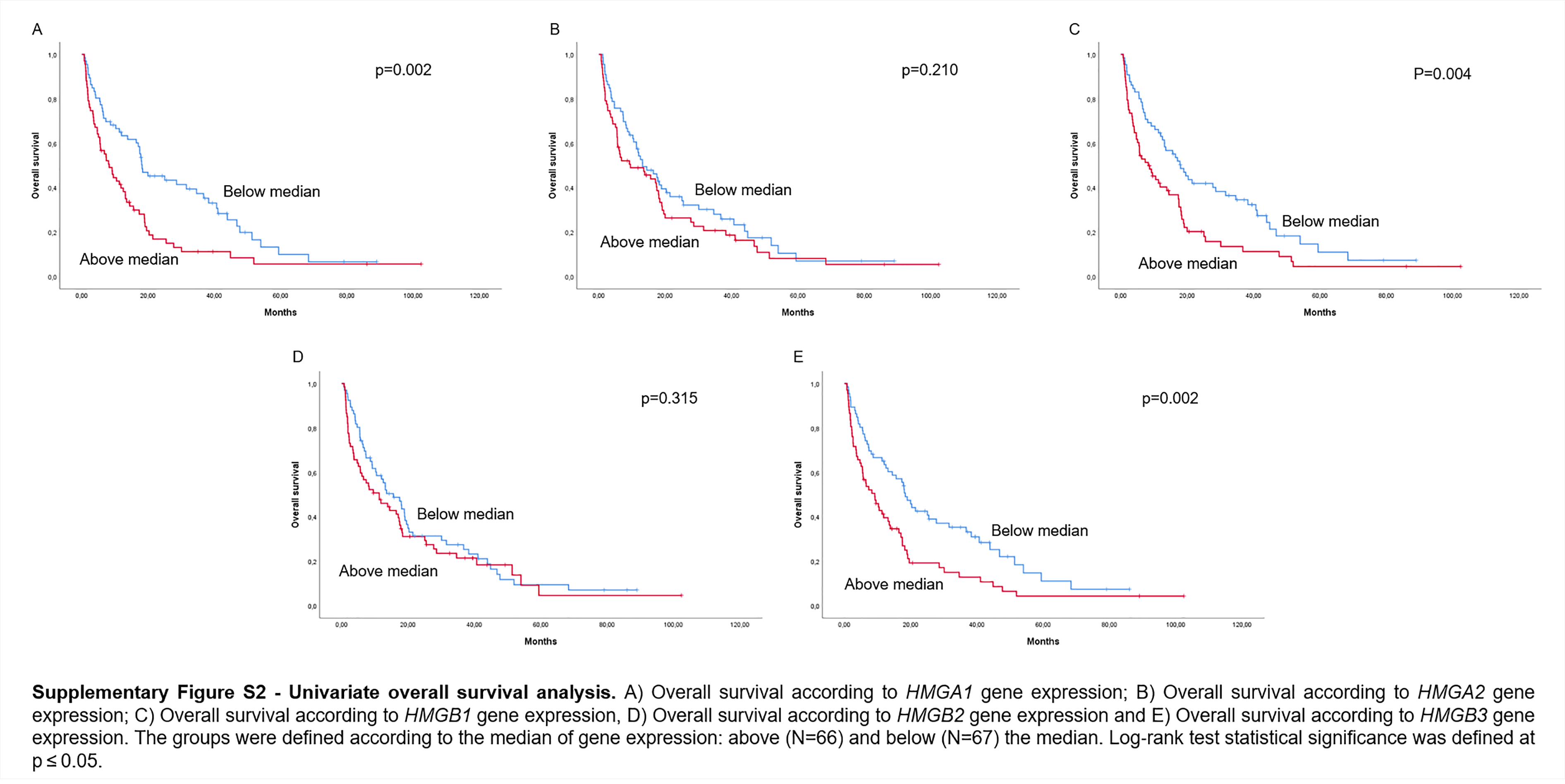

Supplement: Supplementary file 2 — Supplementary file2 (TIF 1189 KB) [file 18_2024_5250_MOESM2_ESM.tif]

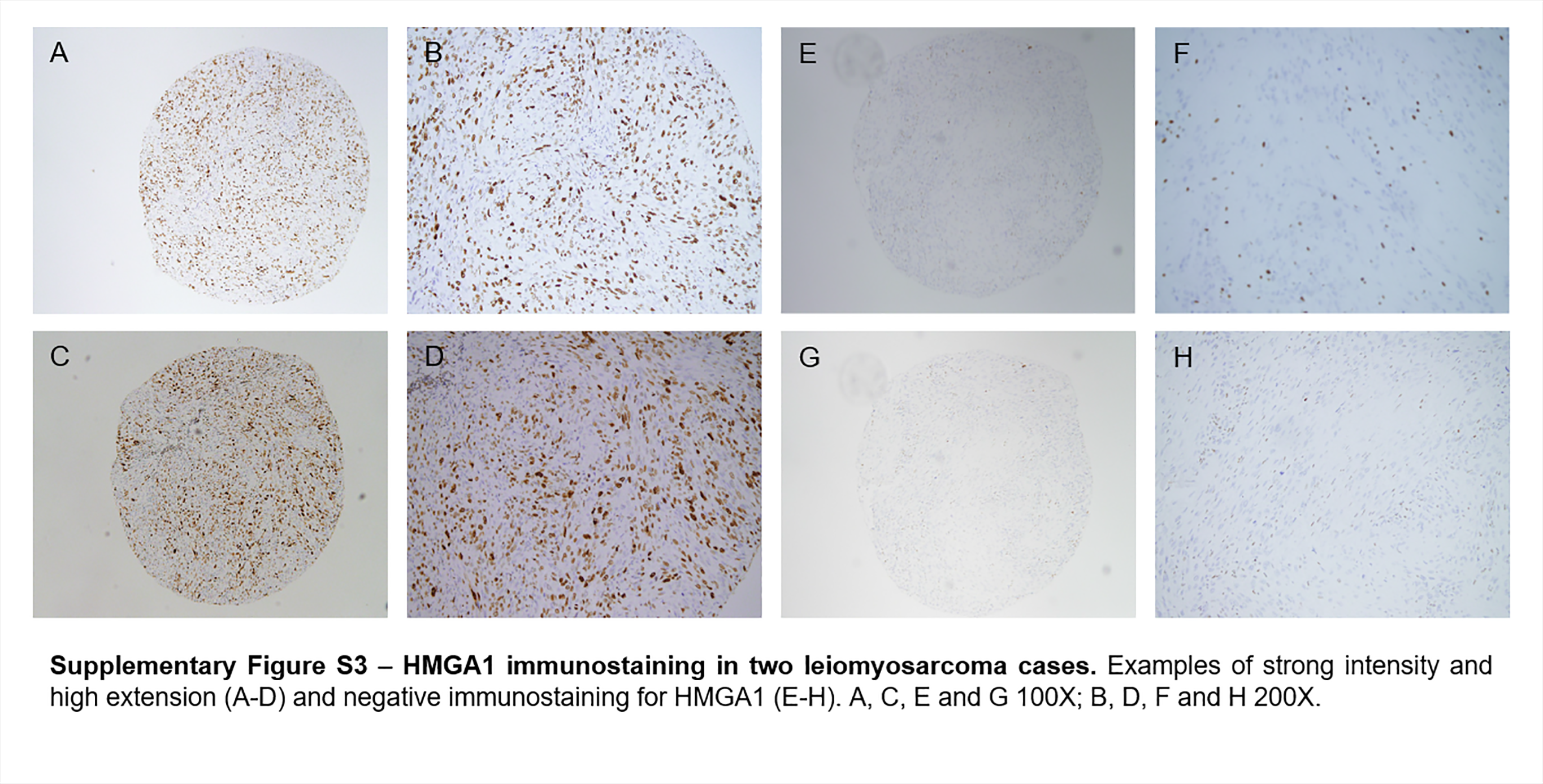

Supplement: Supplementary file 3 — Supplementary file3 (TIF 4527 KB) [file 18_2024_5250_MOESM3_ESM.tif]

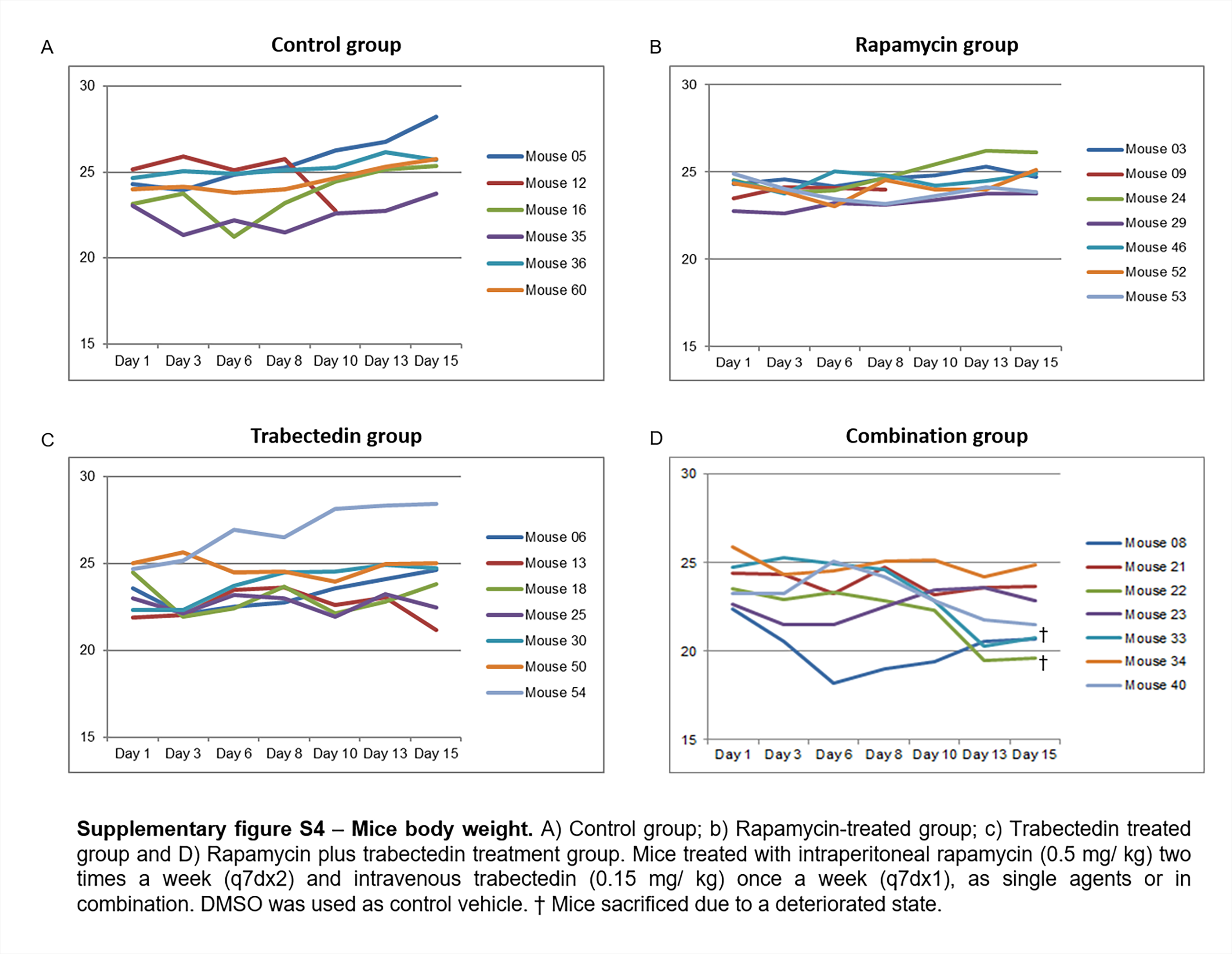

Supplement: Supplementary file 4 — Supplementary file4 (TIF 1522 KB) [file 18_2024_5250_MOESM4_ESM.tif]

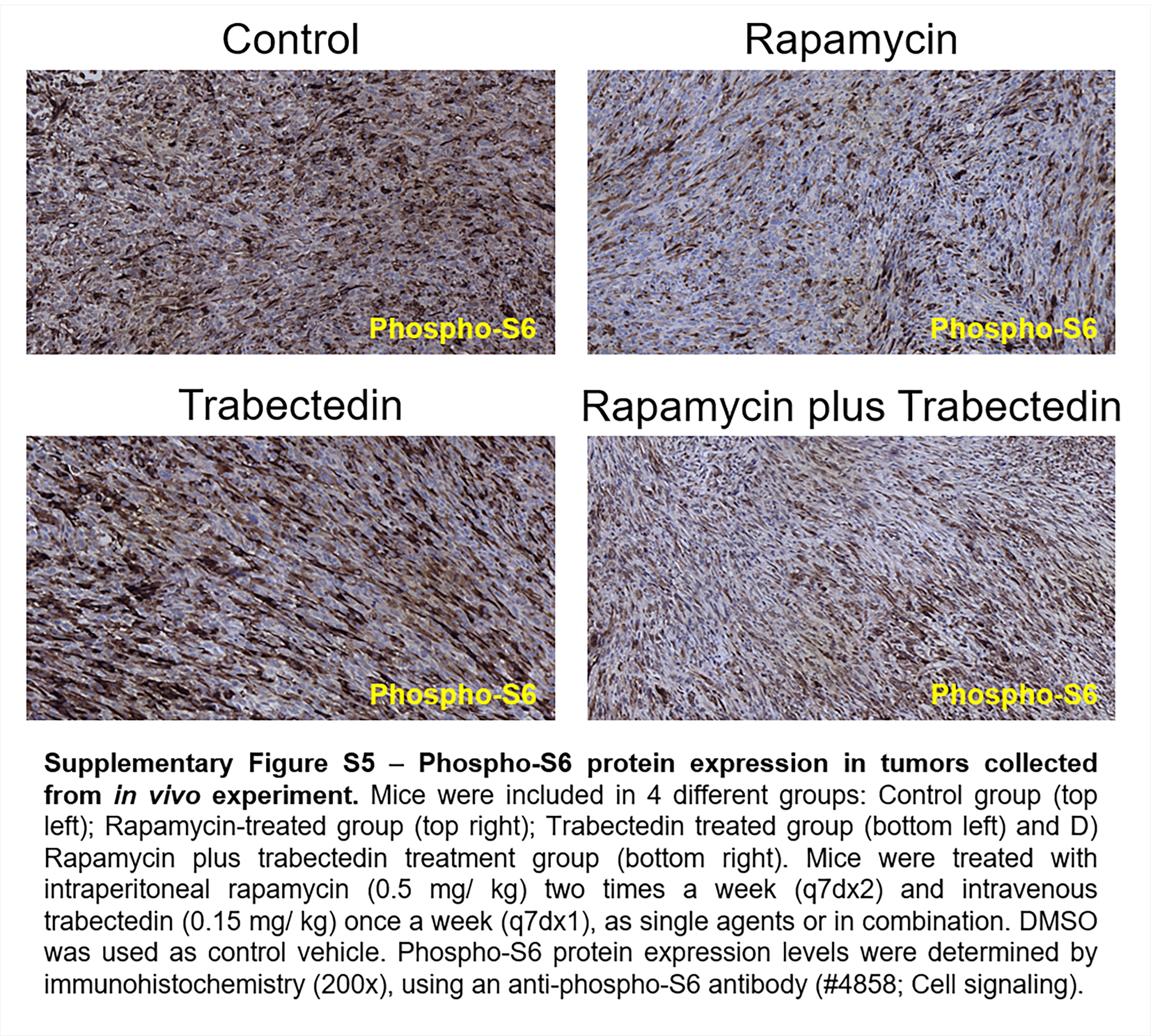

Supplement: Supplementary file 5 — Supplementary file5 (TIF 6334 KB) [file 18_2024_5250_MOESM5_ESM.tif]
