# Supplementary material for: HMGA1 regulates trabectedin sensitivity in advanced soft-tissue sarcoma (STS): A Spanish Group for Research on Sarcomas (GEIS) study
Source: Cell Mol Life Sci. 2024 May 17;81(1):219. doi: 10.1007/s00018-024-05250-y (PMC11101398; doi:10.1007/s00018-024-05250-y)
Supplement: Supplementary file 6 — Supplementary file6 (DOCX 18 KB) [file 18_2024_5250_MOESM6_ESM.docx]

Supplementary Table S1. Univariate analysis of clinical variables

| Factor | PFS (95% CI) | p | OS (95% CI) | p |
| --- | --- | --- | --- | --- |
| Sex:   - Female - Male | 3.4 (2.5-4.3)  3.7 (3.0-4.4) | 0.526 | 13.8 (11.0-16.6)  9.5 (5.7-13.3) | 0.213 |
| Age:   - Below median - Above median | 3.9 (2.5-5.3)  3.2 (2.6-3.8) | 0.028 | 12.9 (8.6-17.3)  10.6 (6.7-14.4) | 0.040 |
| Subtype:   - L-Sarcoma - Non L-Sarcoma | 5.0 (3.4-6.5)  2.6 (2.0-3.2) | <0.001 | 18.8 (13.9-23.6)  6.1 (4.7-7.6) | <0.001 |
| Grade:   - 1 and 2 - 3 | 5.1 (1.9-8.3)  3.0 (2.5-3.5) | <0.001 | 18.3 (13.4-23.2)  8.5 (6.1-10.9) | <0.001 |
| Stage at diagnosis:   - Localized - Metastatic | 3.8 (3.1-4.6)  2.8 (2.3-3.4) | 0.032 | 13.1 (10.0-16.3)  8.0 (3.1-12.8) | 0.309 |
