# Supplementary material for: HMGA1 regulates trabectedin sensitivity in advanced soft-tissue sarcoma (STS): A Spanish Group for Research on Sarcomas (GEIS) study
Source: Cell Mol Life Sci. 2024 May 17;81(1):219. doi: 10.1007/s00018-024-05250-y (PMC11101398; doi:10.1007/s00018-024-05250-y)
Supplement: Supplementary file 7 — Supplementary file7 (DOCX 12 KB) [file 18_2024_5250_MOESM7_ESM.docx]

Supplementary Table S2 – *HMG*s gene expression

| Gene | Median Log2 expression (Range) |
| --- | --- |
| *HMGA1* | 7.46 (5.38 - 12.39) |
| *HMGA2* | 4.57 (-0.46 - 14.30) |
| *HMGB1* | 9.12 (5.99 - 1.54) |
| *HMGB2* | 9.28 (7.06 - 11.58) |
| *HMGB3* | 5.55 (-0.46 - 8.16) |
