# Supplementary material for: HMGA1 regulates trabectedin sensitivity in advanced soft-tissue sarcoma (STS): A Spanish Group for Research on Sarcomas (GEIS) study
Source: Cell Mol Life Sci. 2024 May 17;81(1):219. doi: 10.1007/s00018-024-05250-y (PMC11101398; doi:10.1007/s00018-024-05250-y)
Supplement: Supplementary file 8 — Supplementary file8 (DOCX 14 KB) [file 18_2024_5250_MOESM8_ESM.docx]

Supplementary Table S3. Univariate analysis of HMGs genes for trabectedin survival

| Factor | PFS (95% CI) | p | OS (95% CI) | p |
| --- | --- | --- | --- | --- |
| *HMGA1*   - < 7.46 - ≥ 7.46 | 6.5 (3.9-9.2)  2.7 (1.8-3.5) | 0.001 | 18.2 (10.4-26.0)  8.2 (5.2-11.3) | 0.002 |
| *HMGA2*   - < 4.57 - ≥ 4.57 | 5.4 (3.0-7.7)  2.8 (2.1-3.6) | 0.145 | 13.1 (7.0-19.3)  9.5 (0.0-19.5) | 0.210 |
| *HMGB1*   - < 9.12 - ≥ 9.12 | 6.1 (2.5-9.6)  2.5 (1.7-3.2) | <0.001 | 17.9 (13.0-22.9)  8.2 (3.5-13.0) | 0.004 |
| *HMGB2*   - < 9.28 - ≥ 9.28 | 6.1 (2.6-9.6)  3.1 (2.5-3.7) | 0.013 | 15.6 (9.8-21.3)  11.3 (4.1-18.5) | 0.315 |
| *HMGB3*   - < 5.55 - ≥ 5.55 | 5.6 (1.2-5.4)  2.7 (2.0-3.4) | 0.002 | 18.3 (15.2-21.5)  9.1 (4.9-13.4) | 0.002 |
