# Supplementary material for: HMGA1 regulates trabectedin sensitivity in advanced soft-tissue sarcoma (STS): A Spanish Group for Research on Sarcomas (GEIS) study
Source: Cell Mol Life Sci. 2024 May 17;81(1):219. doi: 10.1007/s00018-024-05250-y (PMC11101398; doi:10.1007/s00018-024-05250-y)
Supplement: Supplementary file 9 — Supplementary file9 (DOCX 12 KB) [file 18_2024_5250_MOESM9_ESM.docx]

Supplementary Table S4 – Multivariate analysis

|  | Progression-free survival | |
| --- | --- | --- |
| Variable | HR  (95% CI) | p |
| *HMGA1* | 1.77  (1.19-2.61) | 0.004 |
| *HMGB1* | 1.59  (1.07-2.37) | 0.023 |
| *HMGB2* | 1.74  (1.19-2.57) | 0.005 |
| L-sarcoma | 0.61  (0.41-0.89) | 0.010 |
