# Supplementary material for: HMGA1 regulates trabectedin sensitivity in advanced soft-tissue sarcoma (STS): A Spanish Group for Research on Sarcomas (GEIS) study
Source: Cell Mol Life Sci. 2024 May 17;81(1):219. doi: 10.1007/s00018-024-05250-y (PMC11101398; doi:10.1007/s00018-024-05250-y)
Supplement: Supplementary file 10 — Supplementary file10 (DOCX 14 KB) [file 18_2024_5250_MOESM10_ESM.docx]

Supplementary Table S5. Univariate analysis of HMGs genes for previous line survival

| Factor | PFS (95% CI) | p | OS (95% CI) | p |
| --- | --- | --- | --- | --- |
| *HMGA1*   - < 7.46 - ≥ 7.46 | 5.6 (4.2-7.0)  4.0 (3.1-4.9) | 0.351 | 33.8 (20.5-47.2)  15.6 (9.6-21.6) | 0.005 |
| *HMGA2*   - < 4.57 - ≥ 4.57 | 5.4 (4.0-6.7)  4.0 (3.3-4.8) | 0.353 | 21.5 (16.1-27.0)  21.4 (17.3-25.4) | 0.511 |
| *HMGB1*   - < 9.12 - ≥ 9.12 | 5.7 (4.8-6.7)  3.9 (3.2-4.6) | 0.074 | 25.4 (13.0-37.8)  14.5 (9.0-19.9) | 0.002 |
| *HMGB2*   - < 9.28 - ≥ 9.28 | 4.4 (3.2-5.6)  5.1 (3.9-6.3) | 0.187 | 23.0 (17.6-28.4)  19.6 (14.4-24.9) | 0.256 |
| *HMGB3*   - < 5.55 - ≥ 5.55 | 5.8 (4.7-6.9)  4.1 (3.4-4.9) | 0.342 | 25.1 (12.0-38.3)  14.5 (8.4-20.6) | 0.023 |
