# Supplementary material for: HMGA1 regulates trabectedin sensitivity in advanced soft-tissue sarcoma (STS): A Spanish Group for Research on Sarcomas (GEIS) study
Source: Cell Mol Life Sci. 2024 May 17;81(1):219. doi: 10.1007/s00018-024-05250-y (PMC11101398; doi:10.1007/s00018-024-05250-y)
Supplement: Supplementary file 11 — Supplementary file11 (DOCX 14 KB) [file 18_2024_5250_MOESM11_ESM.docx]

Supplementary Table S6. Univariate analysis of HMGs proteins for trabectedin survival

| Factor | PFS (95% CI) | p | OS (95% CI) | p |
| --- | --- | --- | --- | --- |
| HMGA1 Expression   - Low (0-49%) - High (50-100%) | 3.9 (2.8-5.0)  2.6 (1.6-3.6) | 0.001 | 13.1 (9.1-17.1)  7.3 (4.9-9.7) | 0.021 |
| HMGA1 Intensity   - Weak-Negative - Strong | 4.0 (3.0-5.0)  2.6 (1.9-3.4) | <0.001 | 13.9 (9.7-18.1)  7.4 (3.7-11.2) | 0.007 |
| HMGB1 Expression   - Low (0-49%) - High (50-100%) | 3.9 (2.4-5.3)  3.4 (2.6-4.1) | 0.454 | 13.9 (10.1-17.7)  12.2 (9.1-15.4) | 0.471 |
| HMGB1 Intensity   - Weak-Negative - Strong | 3.7 (1.9-5.5)  3.4 (2.8-4.1) | 0.223 | 13.9 (8.3-19.6)  11.3 (7.6-15.0) | 0.512 |
