# Supplementary material for: HMGA1 regulates trabectedin sensitivity in advanced soft-tissue sarcoma (STS): A Spanish Group for Research on Sarcomas (GEIS) study
Source: Cell Mol Life Sci. 2024 May 17;81(1):219. doi: 10.1007/s00018-024-05250-y (PMC11101398; doi:10.1007/s00018-024-05250-y)
Supplement: Supplementary file 12 — Supplementary file12 (DOCX 14 KB) [file 18_2024_5250_MOESM12_ESM.docx]

Supplementary Table S7. Univariate analysis of HMGs proteins for trabectedin survival in L-sarcomas

| Factor | PFS (95% CI) | p | OS (95% CI) | p |
| --- | --- | --- | --- | --- |
| HMGA1 Expression   - Low (0-49%)   (N=139)   - High (50-100%)   (N=10) | 5.4 (3.8-6.9)  3.2 (2.1-4.4) | 0.354 | 19.5 (13.9-25.1)  13.9 (2.7-25.0) | 0.126 |
| HMGA1 Intensity   - Weak-Negative   (N=131)   - Strong   (N=18) | 5.6 (4.1-7.1)  3.2 (2.0-4.4) | 0.008 | 21.8 (16.0-27.6)  10.5 (0.8-20.2) | 0.017 |
