# Supplementary material for: HMGA1 regulates trabectedin sensitivity in advanced soft-tissue sarcoma (STS): A Spanish Group for Research on Sarcomas (GEIS) study
Source: Cell Mol Life Sci. 2024 May 17;81(1):219. doi: 10.1007/s00018-024-05250-y (PMC11101398; doi:10.1007/s00018-024-05250-y)
Supplement: Supplementary file 13 — Supplementary file13 (DOCX 14 KB) [file 18_2024_5250_MOESM13_ESM.docx]

Supplementary Table S8. Univariate analysis of HMGs proteins for trabectedin survival in non-L-sarcomas

| Factor | PFS (95% CI) | p | OS (95% CI) | p |
| --- | --- | --- | --- | --- |
| HMGA1 Expression   - Low (0-49%)   (N=85)   - High (50-100%)   (N=38) | 2.7 (2.0-3.4)  2.1 (1.0-3.2) | 0.181 | 6.1 (4.3-7.9)  6.1 (3.5-8.8) | 0.812 |
| HMGA1 Intensity   - Weak-Negative   (N=75)   - Strong   (N=47) | 2.7 (1.9-3.4)  2.6 (1.6-3.6) | 0.421 | 5.7 (3.9-7.4)  7.3 (4.4-10.2) | 0.630 |
