# Supplementary material for: HMGA1 regulates trabectedin sensitivity in advanced soft-tissue sarcoma (STS): A Spanish Group for Research on Sarcomas (GEIS) study
Source: Cell Mol Life Sci. 2024 May 17;81(1):219. doi: 10.1007/s00018-024-05250-y (PMC11101398; doi:10.1007/s00018-024-05250-y)
Supplement: Supplementary file 14 — Supplementary file14 (DOCX 14 KB) [file 18_2024_5250_MOESM14_ESM.docx]

Supplementary Table S9. Univariate analysis of HMGs proteins for trabectedin survival in leiomyosarcoma

| Factor | PFS (95% CI) | p | OS (95% CI) | p |
| --- | --- | --- | --- | --- |
| HMGA1 Expression   - Low (0-49%)   (N=78)   - High (50-100%)   (N=5) | 4.9 (3.1-6.7)  2.5 (1.2-3.9) | 0.008 | 18.2 (13.5-22.9)  13.9 (0.0-35.8) | 0.196 |
| HMGA1 Intensity   - Weak-Negative   (N=71)   - Strong   (N=12) | 5.0 (3.0-6.9)  2.3 (1.6-3.0) | 0.001 | 18.8 (13.5-24.1)  6.6 (0.0-19.7) | 0.228 |
