# Supplementary material for: HMGA1 regulates trabectedin sensitivity in advanced soft-tissue sarcoma (STS): A Spanish Group for Research on Sarcomas (GEIS) study
Source: Cell Mol Life Sci. 2024 May 17;81(1):219. doi: 10.1007/s00018-024-05250-y (PMC11101398; doi:10.1007/s00018-024-05250-y)
Supplement: Supplementary file 15 — Supplementary file15 (DOCX 13 KB) [file 18_2024_5250_MOESM15_ESM.docx]

Supplementary Table S10 – Differential gene expression profile between shHMGA1 and shControl

| **Gene** | **logFC** | **p-value** | **Adjusted p-value** |
| --- | --- | --- | --- |
| *MAGEC2* | -2,091 | <0.001 | <0.001 |
| *DCT* | 1,607 | <0.001 | <0.001 |
| *TNC* | -1,152 | <0.001 | 0.003 |
| *GABRA6* | 1,240 | <0.001 | 0.003 |
| *HSD17B2* | 0,825 | <0.001 | 0.003 |
| *OR51B5* | -0,714 | <0.001 | 0.004 |
| *VAT1L* | 0,937 | <0.001 | 0.005 |
| *MUC13* | 1,079 | <0.001 | 0.006 |
| *CPE* | -0,843 | <0.001 | 0.006 |
| *CSF2RA* | -0,769 | <0.001 | 0.006 |
| *SFXN3* | -0,685 | <0.001 | 0.006 |
| *BTBD3* | -0,608 | <0.001 | 0.009 |
| *KIF5C* | 0,667 | <0.001 | 0.009 |
| *ABI3BP* | 0,992 | <0.001 | 0.009 |
| *SPTLC3* | -1,024 | <0.001 | 0.010 |
| *TPK1* | -0,920 | <0.001 | 0.010 |
| *S1PR1* | 0,837 | <0.001 | 0.010 |
| *ZNF461* | 0,661 | <0.001 | 0.015 |
| *IL1B* | 0,749 | <0.001 | 0.015 |
| *ZNF85* | 0,675 | <0.001 | 0.015 |
| *ETV1* | -0,561 | <0.001 | 0.015 |
| *ADRB2* | -0,700 | <0.001 | 0.020 |
| *TBC1D2* | -0,655 | <0.001 | 0.020 |
| *PLAGL1* | -0,852 | <0.001 | 0.021 |
| *PON2* | -0,489 | <0.001 | 0.021 |
| *PRAME* | -0,510 | <0.001 | 0.023 |
| *FYN* | 0,575 | <0.001 | 0.025 |
| *HBE1* | -0,610 | <0.001 | 0.030 |
| *HDX* | 1,061 | <0.001 | 0.036 |
| *SLC25A35* | -0,593 | <0.001 | 0.040 |
| *CELF2* | 0,742 | <0.001 | 0.043 |
| *CACNB4* | 0,499 | <0.001 | 0.044 |
| *DSEL* | -0,641 | <0.001 | 0.049 |

Positive logFC change means overexpression in shHMGA1 cell cultures
